# Reciprocal interactions among Cobll1, PACSIN2, and SH3BP1 regulate drug resistance in chronic myeloid leukemia

**DOI:** 10.1002/cam4.4727

**Published:** 2022-03-30

**Authors:** Kibeom Park, Hee‐Seop Yoo, Chang‐Kyu Oh, Joo Rak Lee, Hee Jin Chung, Ha‐Neul Kim, Soo‐Hyun Kim, Kyung‐Mi Kee, Tong Yoon Kim, Myungshin Kim, Byung‐Gyu Kim, Jae Sun Ra, Kyungjae Myung, Hongtae Kim, Seung Hun Han, Min‐Duk Seo, Yoonsung Lee, Dong‐Wook Kim

**Affiliations:** ^1^ School of Life Sciences Ulsan National Institute of Science and Technology Ulsan Republic of Korea; ^2^ Department of Molecular Science and Technology Ajou University Suwon Republic of Korea; ^3^ College of Pharmacy and Research Institute of Pharmaceutical Science and Technology College of Pharmacy, Ajou University Suwon Republic of Korea; ^4^ Center for Genomic Integrity Institute for Basic Science Ulsan Republic of Korea; ^5^ Department of Anatomy, School of Medicine Inje University Busan Republic of Korea; ^6^ Leukemia Omics Research Institute Eulji University‐Uijeongbu Campus Gyeonggi‐do Republic of Korea; ^7^ Department of Hematology Catholic Hematology Hospital, Seoul St. Mary's Hospital, College of Medicine, The Catholic University of Korea Seoul Republic of Korea; ^8^ Department of Laboratory Medicine, College of Medicine The Catholic University of Korea Seoul Republic of Korea; ^9^ Department of Medicine Quality Analysis Andong Science College Gyeongbuk Republic of Korea; ^10^ Clinical Research Institute Kyung Hee University Hospital at Gangdong, College of Medicine, Kyung Hee University Seoul Republic of Korea; ^11^ Hematology Center, Uijeongbu Eulji Medical Center Eulji University Gyeonggi‐do Republic of Korea

**Keywords:** blastic transformation, chronic myeloid leukemia, Cobll1, PACSIN2, SH3BP1

## Abstract

Cobll1 affects blast crisis (BC) progression and tyrosine kinase inhibitor (TKI) resistance in chronic myeloid leukemia (CML). PACSIN2, a novel Cobll1 binding protein, activates TKI‐induced apoptosis in K562 cells, and this activation is suppressed by Cobll1 through the interaction between PACSIN2 and Cobll1. PACSIN2 also binds and inhibits SH3BP1 which activates the downstream Rac1 pathway and induces TKI resistance. PACSIN2 competitively interacts with Cobll1 or SH3BP1 with a higher affinity for Cobll1. Cobll1 preferentially binds to PACSIN2, releasing SH3BP1 to promote the SH3BP1/Rac1 pathway and suppress TKI‐mediated apoptosis and eventually leading to TKI resistance. Similar interactions among Cobll1, PACSIN2, and SH3BP1 control hematopoiesis during vertebrate embryogenesis. Clinical analysis showed that most patients with CML have Cobll1 and SH3BP1 expression at the BC phase and BC patients with Cobll1 and SH3BP1 expression showed severe progression with a higher blast percentage than those without any Cobll1, PACSIN2, or SH3BP1 expression. Our study details the molecular mechanism of the Cobll1/PACSIN2/SH3BP1 pathway in regulating drug resistance and BC progression in CML.

## Graphical abstract

Cobll1 preferentially binds to PACSIN2, releasing SH3BP1 to promote the SH3BP1/Rac1 pathway and suppress TKI‐mediated apoptosis and eventually leading to TKI resistance. Clinically, the expression patterns of Cobll1, PACSIN2, and SH3BP1 are highly correlated with severe progression in BC‐CML patients. Our study details the molecular mechanism of the Cobll1/PACSIN2/SH3BP1 pathway in regulating drug resistance in CML and presents a potential pharmacological target to overcome TKI resistance and BC progression in CML.

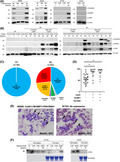

## 1 INTRODUCTION

Chronic myeloid leukemia (CML) is a malignant clonal disorder of hematopoietic stem cells^1^ that progresses through three clinical phases: the chronic phase (CP), accelerated phase (AP), and blast crisis (BC).^2^ The major cause of CML initiation is a translocation that results in the BCR‐ABL1 fusion gene.^3^ Constitutive expression of the BCR‐ABL1 fusion tyrosine kinase enhances genomic instability. The BCR‐ABL1 tyrosine kinase inhibitor (TKI) imatinib has been shown to be highly efficacious in CML treatment. However, imatinib is unable to completely eliminate CML clones due to the development of resistance and the insensitivity of primitive CML cells to the treatment.^4^, ^5^ Despite the development of more potent next‐generation BCR‐ABL1 TKIs including ATP and allosteric site inhibitors,^6^, ^7^, ^8^, ^9^ some patients still develop TKI resistance during BC progression in both a BCR‐ABL1 kinase‐dependent or ‐independent manner.^10^

Cobll1, a negative regulator of apoptosis, is associated with lower insulin resistance.^11^, ^12^ Patients with CML and chronic lymphocytic leukemia (CLL) possessing high Cobll1 expression have shown poor clinical outcomes.^13^, ^14^ Previously, we demonstrated that Cobll1 leads to the induction of TKI resistance and blastic transformation in CML.^13^ In addition, Plešingerová et al. reported that patients with CLL and high Cobll1 expression but without the *IGHV* mutation exhibit unregulated B‐cell receptor signaling, impaired migration, and chemotaxis.^14^ However, the underlying molecular mechanisms of how Cobll1 expression represses apoptotic cell death resulting in TKI resistance in CML are not fully understood.

Protein kinase C and casein kinase substrate in neurons 2 (PACSIN2) are a ubiquitously expressed cytoplasmic adapter protein.^15^ It possesses an N‐terminal F‐BAR domain and a C‐terminal Src homology 3 (SH3) domain. PACSIN2 is known to function in cellular migration,^16^ caveolae shaping,^17^, ^18^, ^19^ and receptor internalization.^20^, ^21^ PACSIN2 interacts with several endocytic proteins, including dynamin 2, neural Wiskott–Aldrich syndrome protein, and synaptojanin, through its SH3 domain.^22^, ^23^ Clinically, PACSIN2 polymorphism has been shown to be linked to drug‐induced hematological toxicity in patients with acute lymphoblastic leukemia (ALL) undergoing therapy.^24^, ^25^

SH3 domain‐binding protein 1 (SH3BP1) is a RhoGTPase‐activating protein that is important for the process of cell motility and cancer invasion through the regulation of the Rac1 pathway.^26^ Overexpression of SH3BP1 activates the invasion and migration of cancer cells by increasing the activity of Rac1‐WAVE2 signaling in cervical cancer and hepatocellular carcinoma.^27^, ^28^ Rac1 has been identified as an important downstream component of BCR‐ABL1 signaling and a key therapeutic target for CML.^29^, ^30^ Activation of the Rac1 pathway induces TKI resistance, whereas Rac1 inhibition reduces the proliferation of erythroid progenitor cells resistant to TKI.^31^

Here, we identified PACSIN2 as a novel binding partner of Cobll1. PACSIN2 promoted nilotinib‐induced apoptosis, which was suppressed by Cobll1 expression. Additionally, PACSIN2 binds to another SH3 domain‐binding protein, SH3BP1, thereby repressing the SH3BP1/Rac1 signaling cascade. Cobll1 preferentially competes with SH3BP1 for binding to PACSIN2, eventually releasing SH3BP1 from PACSIN2 to suppress nilotinib‐dependent apoptosis by inducing the Rac1 pathway. Likewise, reciprocal interaction among *cobll1b*, *pacsin2*, and *sh3bp1* regulates hematopoiesis during embryonic development in zebrafish. Furthermore, analysis of large sets of primary CML samples showed that the expression patterns of Cobll1, PACSIN2, and SH3BP1 were highly correlated with the BC progression. Taken together, our studies showed that reciprocal interaction between Cobll1, PACSIN2, and SH3BP1 is closely linked to drug resistance and BC progression in CML. Our results suggest that the pharmacologic targeting of the Cobll1/PACSIN2/SH3BP1 axis can be a novel approach to overcome TKI resistance and disease progression in CML.

## 2 MATERIALS AND METHODS

### 2.1 Patient samples and cell lines

A total of 100 CML samples (36 CP including NEL and CHR samples, four AP, and 60 BC) from bone marrow (BM) and peripheral blood (PB) were obtained from 57 CML patients. Among them, 10 patients had serial samples at CP and BC. Mononuclear cells (MCs) were isolated by Ficoll‐Paque (GE Healthcare, Waukesha, WI, USA) density gradient centrifugation. Samples were frozen in 10% dimethyl sulfoxide (DMSO; Sigma‐Aldrich, St. Louis, MO, USA) in fetal bovine serum (Sigma‐Aldrich), stored in liquid nitrogen, and later thawed for analysis. All human samples were obtained from the Korea Leukemia Bank and the protocol was approved by the Institutional Review Board. Patient consent was obtained in accordance with the Declaration of Helsinki.

### 2.2 Plasmids

The full‐length cDNA clone for Cobll1 (Clone ID: 30345650) was purchased from Thermo Scientific (Waltham, MA, USA). The full‐length cDNA clone for PACSIN2 (Clone ID: hMU008023) was purchased from the Korea Human Gene Bank. The Myc‐PACSIN2, SFB‐PACSIN2, Myc‐SH3BP1, and SFB‐SH3BP1 internal deletion mutants were generated by polymerase chain reaction (PCR). The full‐length cDNA clone for Rac1 (Clone ID: hMU007586) was purchased from the Korea Human Gene Bank. The wild‐type and active forms of HA‐Rac1 were generated by PCR. GST‐SH3 was also generated by PCR.

### 2.3 Immunoprecipitation and western blot

K562 cells were transfected with expression plasmids, as indicated in each figure legend. After 24 h, the cells were lysed in NETN buffer (150 mM NaCl, 0.5 mM EDTA, 50 mM Tris, pH 7.5, 0.5% NP‐40) for 20 min on ice. The crude lysates were cleared by centrifugation at 13,000 rpm for 15 min at 4°C and the supernatants were incubated with streptavidin‐coated beads or protein A‐agarose‐conjugated primary antibodies. The resulting immunocomplexes were washed three times with NETN buffer and subjected to sodium dodecyl sulfate‐polyacrylamide gel electrophoresis (SDS–PAGE). Western blotting was conducted using the antibodies indicated in the figure legends.

### 2.4 Antibodies and dilution factors

The dilutions of the various antibodies for western blot analysis are as follows: anti‐Cobll1, which was previously described, 1:200; anti‐PACSIN2 (Sigma‐Aldrich, SAB1300127), 1:1000; anti‐FLAG (Sigma‐Aldrich, F3165), 1:2000; anti‐Myc (Roche, 11,814,150,001) (Roche, Basel, Switzerland), 1:2000; anti‐HA (Roche, 12,013,819,001), 1:2000; anti‐β‐actin (Sigma‐Aldrich, A5316), 1:5000; anti‐GFP (Clontech 632,380) (Clontech, Mountain View, CA, USA), 1:2000; anti‐SH3BP1 (Proteintech, 20,541‐1‐AP), 1:1000. Horseradish peroxidase‐conjugated secondary antibodies specific to rabbit (Sigma‐Aldrich, A0545) or mouse (Sigma‐Aldrich, A9917) IgG were used at a dilution of 1:2000.

### 2.5 Cell culture

The K562 cell lines were maintained in RPMI medium supplemented with 10% fetal bovine serum (Gibco, Franklin Lakes, NJ) and 1% penicillin/streptomycin (Gibco) at 37°C in a 5% CO_2_ atmosphere. The human embryonic kidney 293 T (HEK293T) cell lines were maintained in DMEM supplemented with 10% fetal bovine serum and 1% penicillin/streptomycin at 37°C in a 5% CO_2_ atmosphere.

### 2.6 Apoptosis assay

The plasmids or siRNAs were transfected into K562 cells, as indicated in the figures. After 48 h, the transfected cells were seeded in 100‐mm culture dishes and treated with the indicated concentrations of nilotinib for 48 h. The cells were then assayed for apoptosis using the FITC Annexin V Apoptosis Detection Kit I (BD Bioscience, 556,547, San Jose, CA, USA) according to the manufacturer's protocol. In addition, the apoptotic cells were analyzed by fluorescence‐activated cell sorting (FACS) (BD Bioscience). Nilotinib (Sigma‐Aldrich) was reconstituted in DMSO as a 10 mM stock and stored at −20°C.

### 2.7 Protein purification

Genes encoding the N‐terminal regions of Cobll1 (residues 1–410; Cobll1‐NT‐f and 175–370; Cobll1‐NT), PACSIN2 SH3 domain (residues 430–486), and C‐terminal region of SH3BP1 (residues 541–701; SH3BP1‐CT) were cloned into the pGEX‐4 T‐1 and pET‐21a vectors, respectively. The recombinant vectors were transformed into *E. coli* BL21‐CodonPlus (DE3) cells, and the transformed cells were grown in LB medium and induced with 0.5 mM isopropyl β‐D‐1‐thiogalactopyranoside (IPTG). The cells were harvested and disrupted by sonication in lysis buffer (20 mM Tris–HCl, 500 mM NaCl, 10% glycerol, 0.2% Nonidet P‐40 (NP40), 0.4 mM Tris(2‐carboxyethyl) phosphine hydrochloride (TCEP), 1 mM EDTA, pH 7.0). For the GST‐fusion proteins (Cobll1‐NT‐f, Cobll1‐NT, and PACSIN2 SH3 domain), the proteins were loaded onto a GST column and eluted with elution buffer (20 mM Tris–HCl, 200 mM NaCl, 5% glycerol, 10 mM DTT, 0.2 mM TCEP, 1 mM EDTA, 10 mM glutathione, pH 7.0). The GST tags were cleaved by PreScission protease (Thermo Fisher Scientific) or thrombin and the cleaved proteins were purified by ion exchange chromatography using stepwise elution with increasing ionic strength (up to 1 M NaCl). Further purification was performed using a HiLoad 16/600 Superdex 75/200 prep grade column (GE Healthcare) with final buffer (50 mM sodium phosphate, 100 mM NaCl, 1 mM TCEP, pH 6.3). For poly‐histidine‐tagged proteins (SH3BP1‐CT), the proteins were loaded onto a Ni column and eluted by increasing concentrations of imidazole (up to 1 M imidazole). Further purification was performed using a HiLoad 16/600 Superdex 75 prep grade column (GE Healthcare).

### 2.8 Surface plasmon resonance (SPR)

All SPR experiments were conducted at 25°C in PBS buffer (10 mM KH_2_PO_4_, 137 mM NaCl, and 2.7 mM KCl, pH 7.4). The PACSIN2 SH3 domain was immobilized on a PEG chip using amine coupling in the Reichert SR7500DC system (Reichert Technologies Life Sciences). The flow rate for ligand immobilization was 10 μl/min for 20 μg of PACSIN2 SH3 domain on the sample channel. The N‐terminal region of Cobll1 (residues 1–410; Cobll1‐NT‐f) and the C‐terminal region of SH3BP1 (residues 541–701; SH3BP1‐CT) were dissociated for 10 min after 3 min of association time. The sensor surface was regenerated with 100 mM NaOH solution between assays. The binding curves were analyzed using Scrubber2 software (BioNavis).

### 2.9 NMR spectroscopy

The [^15^N]‐ or [^15^N, ^13^C]‐labeled PACSIN2 SH3 domain was purified with the same procedures. Backbone assignments were performed with ^1^H‐^15^N Heteronuclear single quantum coherence (HSQC) recorded at 298 K on a Bruker AVANCE II 600 MHz spectrometer (Gachon University, Incheon). All ^1^H‐^15^N HSQC NMR titrations were performed at 298 K on a Bruker AVANCE II 800 MHz spectrometer (Korea Basic Science Institute, O‐Chang). For Cobll1‐NT and SH3PB1‐CT titrations, unlabeled Cobll1‐NT and SH3BP1‐CT proteins were purified and prepared in the same buffer (50 mM sodium phosphate, 100 mM NaCl, 1 mM TCEP, pH 6.3). The proteins were titrated to 50 μM of ^15^N‐labeled PACSIN2 SH3 domain at various molar ratios (PACSIN2 SH3 domain: Cobll1‐NT:SH3BP1‐CT = 1:0:0, 1:0:1, 1:1:0, 1:1:1). All spectra were processed using NMRpipe and visualized using NMRViewJ software.

### 2.10 MO and mRNA injection

Wild‐type zebrafish of AB* strain were raised in an automatic circulating system (Genomic‐Design) at 28.5°C and maintained in accordance with guidelines of the Ulsan National Institute of Science and Technology (UNIST) Institutional Animal Care and Use Committee (IACUC) (IACUC approval number: UNISTIACUC‐15‐14). Every MO against *cobll1b*, *pacsin2*, *sh3bp1*, and *rac1a* (Gene Tools, Philomath, OR, USA) was diluted in diethyl pyrocarbonate (DEPC)‐treated water as a 25 ng/nL stock. Ten nanograms of cobll1b‐MO, 1 ng of pacsin2‐MO, 2.5 ng of sh3bp1‐MO, and 2.5 ng of rac1a‐MO were injected into wild‐type embryos at the one‐cell stage. The sequences of the splice‐blocking MOs were 5′‐CTTCTCCAATCAGTCGCCCTCAC‐3′ for pacsin2‐MO, 5′‐ATCATTCAAACCTTCACCTTATAGA‐3′ for sh3bp1‐MO, and 5′‐CATCTGTATTAGCTTGTTACCGTCT‐3′ for rac1a‐MO. The cDNA of *pacsin2* was synthesized using wild‐type zebrafish embryo RNA and inserted into the PCS2+ vector. Full‐length *pacsin2* was synthesized from linearized pCS2+ *pacsin2* through in vitro transcription (mMESSAGE mMACHINE Kit, Ambion). *Pacsin2* mRNA was injected into wild‐type embryos at the one‐cell stage. Microinjections were performed using a Femtojet 4i microinjector (Eppendorf).

## 3 RESULTS

### 3.1 Identification of PACSIN2 as a novel Cobll1 binding protein

**FIGURE 1:**
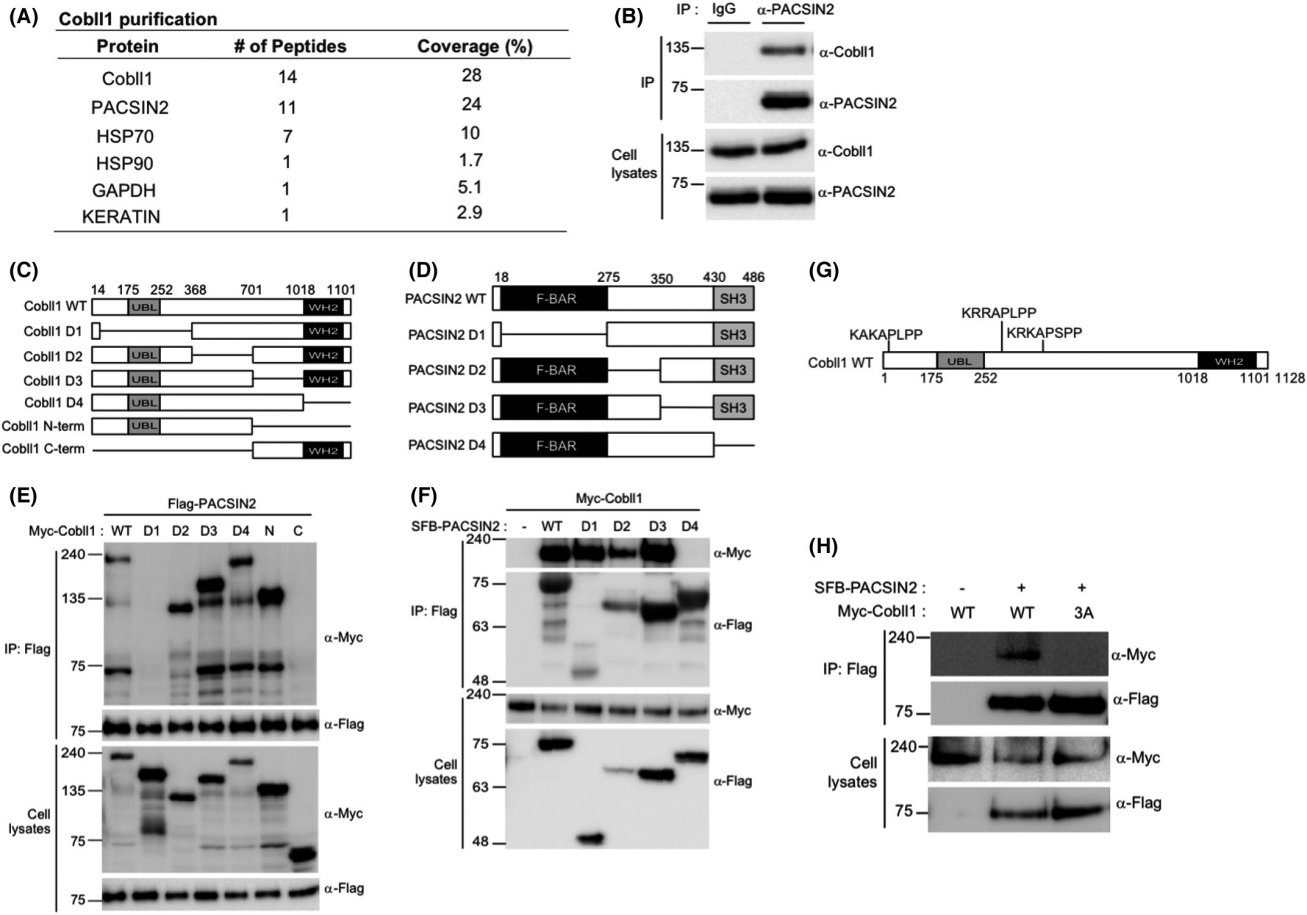
PACSIN2 is a new binding protein of Cobll1. (A) List of proteins identified that interact with Cobll1 by mass spectrometry analysis. (B) Western blot demonstrating interaction between endogenous Cobll1 and PACSIN2 in K562 cells. (C and D) Diagram of wild‐type (WT) Cobll1 or PACSIN2 and their internal deletion mutants. Residue numbers are shown above the diagram. (E and F) Identification of interacting domains of Cobll1 and PACSIN2. (G) Diagram describing three primary proline‐rich regions in Cobll1. (H) Interaction between PACSIN2 and overexpressed Cobll1‐WT or Cobll1‐3A (3A) wherein all three proline‐rich motifs of Cobll1 have been mutated to alanine

Previously, we reported that Cobll1 affects nilotinib sensitivity in CML and regulates embryonic hematopoiesis.^13^ To further investigate the molecular function of Cobll1, we performed tandem affinity purification and a yeast two‐hybrid screening of a human bone marrow cDNA library with Cobll1 as a bait (Figure S1A). Mass spectrometric analysis identified several binding protein candidates, and 14 positive clones were recovered from the yeast two‐hybrid screening analysis (out of a total of 4.5 × 10^6^ transformants). PACSIN2 was identified as the most promising Cobll1‐interacting protein with 24% of the peptides in the proteomic analysis (Figure 1A). Additionally, 11 out of 14 clones in the yeast two‐hybrid analysis were derived from PACSIN2 (Figure S1B). Furthermore, we confirmed the interaction between Cobll1 and PACSIN2 through co‐immunoprecipitation using both endogenous and overexpressed Cobll1 and PACSIN2 (Figure 1B; Figure S1C,D).

To identify essential interaction domains in Cobll1 and PACSIN2, we expressed deletion mutants of the Cobll1 and PACSIN2 proteins in K562 cell lines and probed the interaction by co‐immunoprecipitation (Figure 1C,D). We found that deletion of amino acids 14–368 of Cobll1 abolished its interaction with PACSIN2 (Figure 1E), whereas amino acids 430–486 of PACSIN2, which contained the SH3 domain, were essential for the interaction with Cobll1 (Figure 1F). The interaction between the SH3 domain of PACSIN2 and Cobll1 was further confirmed using a GST pull‐down assay (Figure S2A). Because the SH3 domain generally interacts with proline‐rich motifs in their respective binding partner,^32^ we analyzed the availability of proline‐rich motifs in Cobll1 using the modular domain peptide interaction (modpepint) server.^33^ We found that Cobll1 possesses three proline‐rich motifs in its N‐terminus (Figure 1G; Figure S2B). To validate the roles of each proline‐rich motif on the binding to the SH3 domain of PACSIN2, we generated mutants in which the proline residues in one, two, or all three motifs were mutated to alanine (Figure S2C). Mutating all proline residues (Cobll1‐3A mutant) led to an almost complete loss of interaction with PACSIN2, whereas mutations of two motifs (Cobll1‐2A mutants) or only one proline motif (Cobll1‐1A mutants) showed no or little effect on binding compared to that of the wild‐type protein (Figure S2D). We further examined the role of the proline‐rich motifs of Cobll1 on interaction with PACSIN2 by co‐immunoprecipitating Cobll1 wild‐type or Cobll1‐3A mutants with PACSIN2 and found that Cobll1 failed to bind to PACSIN2 without proline‐rich motifs (Figure 1H). Taken together, these results indicate that Cobll1 strongly binds to the SH3 domain of PACSIN2 through its proline‐rich motifs.

### 3.2 Interaction between Cobll1 and PACSIN2 regulates nilotinib resistance

**FIGURE 2:**
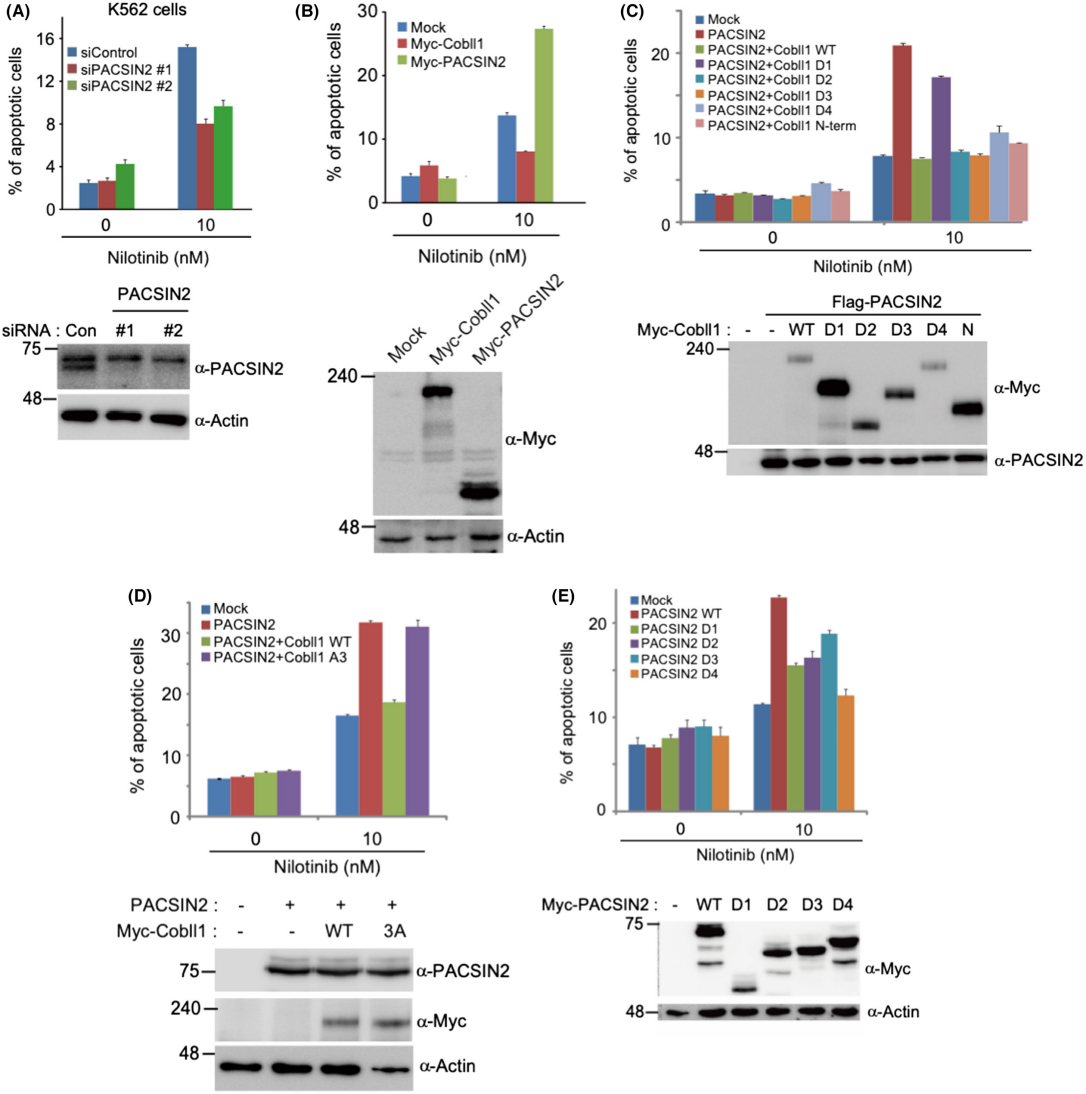
PACSIN2 regulates nilotinib resistance in K562 cells. (A and B) Quantification of nilotinib‐induced apoptotic events with knockdown of PACSIN2 (A) and overexpression of PACSIN2 and Cobll1 (B). Bar graph illustrating the percentage of apoptotic cells. (C‐E) Percentage of nilotinib‐induced apoptosis with the expression of the indicated genes; co‐expression of full‐length PACSIN2 with distinct deleted forms of Cobll1 (C), co‐expression of full‐length PACSIN2 and the Cobll1‐3A mutant with mutations in the proline‐rich regions (D), and expression of the deleted forms of PACSIN2 (E). Bar graphs showing the average of three independent experiments, with error bars indicating the SEM. Expression of Cobll1 and PACSIN2 was confirmed by western blotting images provided below each graph

Due to the effects of Cobll1 on drug sensitivity in CML, we further validated the function of PACSIN2 on nilotinib resistance by evaluating the apoptotic index using fluorescence‐activated cell sorting (FACS) analysis (Figure S3). First, the depletion of PACSIN2 using siRNAs in K562 cells yielded lower nilotinib‐induced apoptosis than in the controls (Figure 2A). Knockdown efficiency of PACSIN2 siRNA without off target effects was confirmed by the rescue of siRNA effects through the expression of siRNA‐resistant PACSIN2 (Figure S4). PACSIN2 overexpression increased apoptosis by approximately twofold, whereas Cobll1 overexpression decreased nilotinib‐induced apoptosis (Figure 2B). Moreover, cells expressing both PACSIN2 and Cobll1 had less apoptosis than PACSIN2‐overexpressing cells, suggesting that reciprocal interaction between PACSIN2 and Cobll1 is crucial for regulating TKI sensitivity (Figure 2C). Thus, we further examined whether the direct interaction between Cobll1 and PACSIN2 influences nilotinib‐induced apoptosis in CML. While the expression of full‐length Cobll1 reduced PACSIN2‐induced apoptosis to the control level, overexpression of a PACSIN2‐interaction‐defective form of Cobll1 protein D1 (described in Figure 1C) was dispensable for the apoptotic events induced by PACSIN2 overexpression (Figure 2C). Consistently, nilotinib sensitivity was not restored by the overexpression of the Cobll1‐3A mutant (Figure 2D). In contrast to the D1 form and the Cobll1‐3A mutant, other deleted forms of Cobll1 (D2, D3, D4, and N‐term, all of which can bind to PACSIN2), recovered cell death to the control level in the presence of PACSIN2 expression. This result suggests that Cobll1 represses PACSIN2‐induced apoptosis with nilotinib treatment via physical interaction between the two proteins.

To further validate the role of the PACSIN2‐Cobll1 interaction, we examined the function of the interaction‐defective PACSIN2 with Cobll1 for nilotinib sensitivity in K562 cells. Surprisingly, overexpression of the PACSIN2 D4 mutant, which lacks the SH3 domain required for Cobll1 binding (Figure 1D), led to no significant alteration in cell death in K562 cells with nilotinib stimulation. By contrast, wild‐type or D1/D2/D3 mutants, which possess an interaction domain for Cobll1, induced apoptosis (Figure 2E). Taken together, these results demonstrate that the SH3 domain of PACSIN2 is essential for inducing apoptosis with TKI treatment and for binding to Cobll1, which suppresses the PACSIN2‐dependent apoptotic process.

### 3.3 Identification of SH3BP1 as a novel PACSIN2 binding protein

**FIGURE 3:**
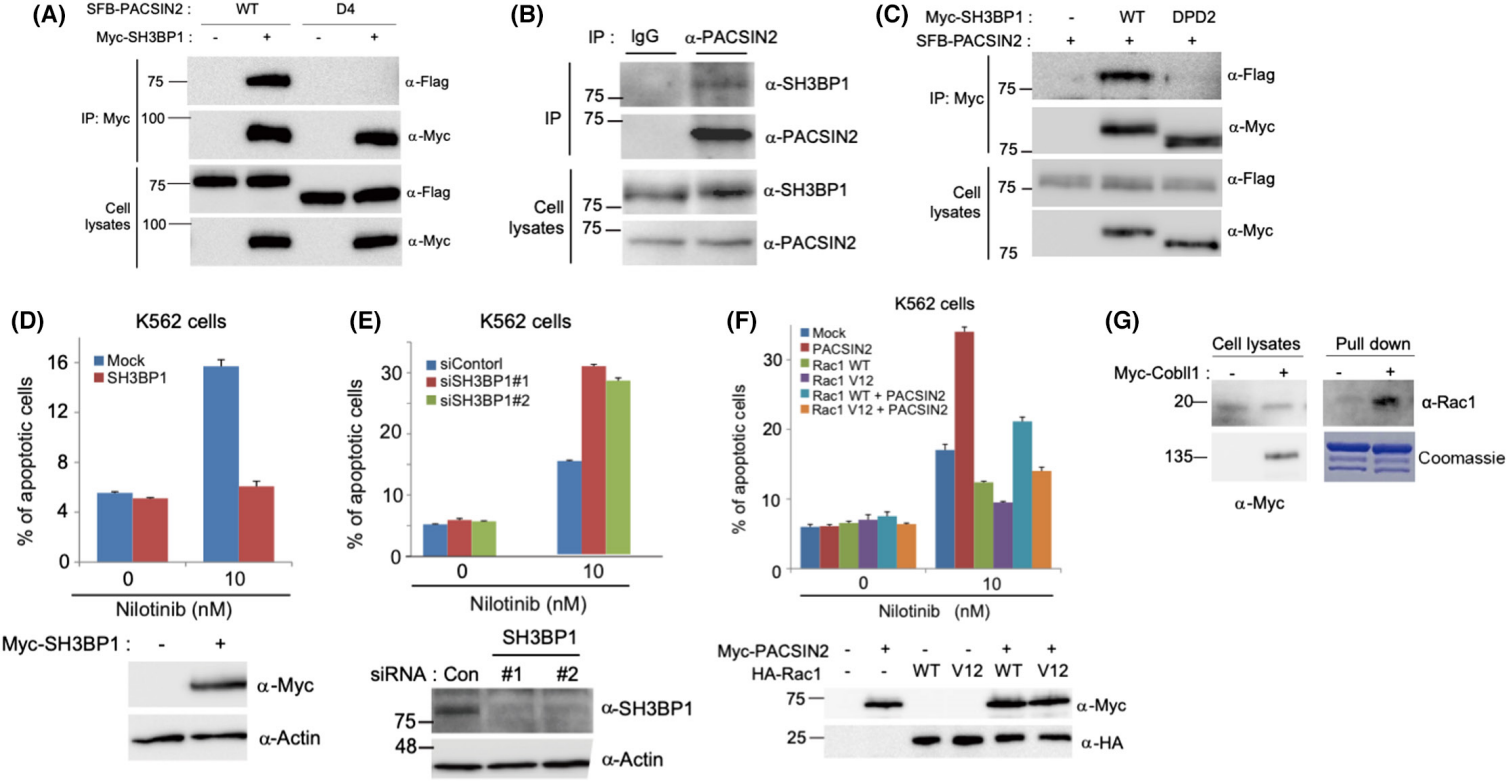
The PACSIN2 binding protein SH3BP1 induces drug resistance in K562 cells. (A) Co‐immunoprecipitation assay using full‐length PACSIN2 and PACSIN2 D4 mutant with the SH3BP1 protein. (B) Immunoprecipitation assay using endogenous PACSIN2 and SH3BP1 proteins. (C) Interaction between the overexpressed SH3BP1‐DPD2 mutant and PACSIN2. SH3BP1‐DPD2 indicates a deletion mutant wherein all five proline‐rich motifs of SH3BP1 have been deleted. (D‐F) Quantification of nilotinib‐induced apoptosis with overexpression of SH3BP1 (D), knockdown of SH3BP1 using siRNA (E), and overexpression of PACSIN2 and Rac1 in K562 cells (F). (G) GST pull‐down assay using GST‐Rac1 and cell lysates transfected with or without Cobll1 expression plasmid

According to our experimental results, the Cobll1‐binding SH3 motif of PACSIN2 is essential for the function of PACSIN2 to promote TKI‐induced apoptosis, as well as for the inhibition of this function through Cobll1 binding. Therefore, we hypothesized that another binding partner for the SH3 domain of PACSIN2 could be involved in the nilotinib‐induced apoptotic process in CML. To find the candidates, we searched for binding proteins using tandem affinity purification with PACSIN2 and found the following potential PACSIN2‐binding proteins: Cobll1, SH3BP1, ARHGAP17, TCOF1, DNM2, and WIPF2 (Figure S5A,B). Because SH3BP1 contains multiple proline‐rich regions, which can potentially bind to the SH3 domain (Figure S5C), we first examined whether SH3BP1 can interact with PACSIN2 by binding to the SH3 motif. SH3BP1 was co‐immunoprecipitated by full‐length PACSIN2 but not by the PACSIN2 D4 mutant lacking the SH domain, suggesting that the interaction requires the SH3 domain of PACSIN2 (Figure 3A). We confirmed the interaction between PACSIN2 and SH3BP1 through an immunoprecipitation assay using endogenous proteins and a GST pull‐down assay (Figure 3B; Figure S5D).

To closely dissect the PACSIN2 binding regions on SH3BP1, we analyzed possible proline‐rich motifs of SH3BP1 using the MoDPepInt server, the Eukaryotic Linear Motif resource, and BLAST^33^, ^34^ and found five potential motifs within SH3BP1 (Figure S5C,E). To identify which proline‐rich motif of SH3BP1 is required for PACSIN2 binding, we generated SH3BP1 mutant proteins with deletions or point mutations incorporated into each proline‐rich motif (Figure S5F). GST pull‐down assays showed that the SH3BP1 DPD2 mutant, wherein all five available proline‐rich motifs were mutated, failed to interact with the PACSIN2 SH3 domain. All the other SH3BP1 mutants possessing at least one proline‐rich motif were bound to the SH3 domain of PACSIN2 (Figure S5G). Moreover, PACSIN2 was co‐immunoprecipitated with wild‐type SH3BP1 but not with the DPD2 mutant (Figure 3C). Collectively, our data indicate that the proline‐rich motifs of SH3BP1 are crucial for its interaction with PACSIN2 and that each motif is sufficient to interact with the SH3 domain of PACSIN2.

### 3.4 SH3BP1 induces nilotinib resistance

The RhoGTPase‐activating protein SH3BP1 activates Rac1‐WAVE2 signaling in diverse types of cancer.^27^, ^28^ Rac1 is well known for its function related to TKI resistance and as a key component of the therapeutic target for CML.^31^ To identify whether SH3BP1 and downstream Rac1 signaling are involved in drug sensitivity in CML, we investigated the role of SH3BP1 and Rac1 in nilotinib resistance using the K562 cell line. In contrast to PACSIN2, SH3BP1 overexpression decreased nilotinib‐induced apoptosis by approximately threefold (Figure 3D), while SH3BP1 depletion using siRNA increased apoptosis rate by approximately twofold (Figure 3E). Similarly, overexpression of wild‐type Rac1 or the active form of Rac1 (Rac1 V12) alone reduced nilotinib‐induced apoptosis while PACSIN2 expression restored the apoptosis level in the presence of Rac1 (Figure 3F). Moreover, Cobll1 overexpression, which suppresses PACSIN2‐dependent apoptosis with nilotinib treatment, subsequently activated Rac1 (Figure 3G). Taken together, these results suggest that SH3BP1/Rac1 signaling induces CML drug resistance, which can be repressed by PACSIN2.

### 3.5 Cobll1 and SH3BP1 competes for binding to PACSIN2

**FIGURE 4:**
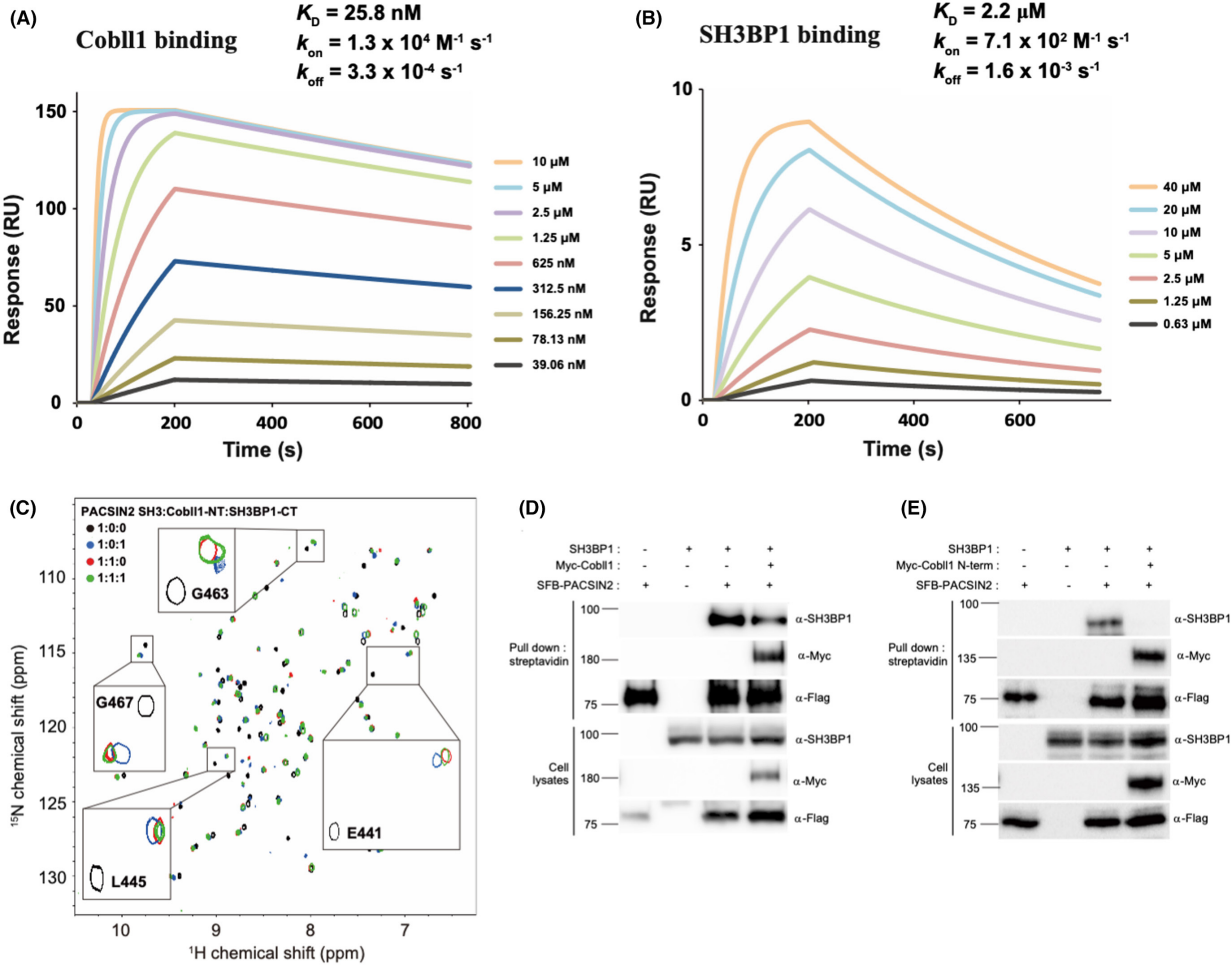
Cobll1 and SH3BP1 competes for binding to the SH3 domain of PACSIN2. (A and B) Surface plasmon resonance (SPR) analysis of the PACSIN2 SH3 domain binding to the N‐terminal region of Cobll1 (A) and the C‐terminal region of SH3BP1 (B). (C) Heteronuclear single quantum coherence (HSQC) spectroscopy spectra of the PACSIN2 SH3 domain in the presence of SH3BP1‐CT (blue), Cobll1‐NT (red), and both proteins (green). (D and E) Western blot images showing that both the full‐length (D) and N‐terminal region (E) of Cobll1 interrupt binding between PACSIN2 and SH3BP1. All graphs show the percentage of apoptotic cells as the average of three independent experiments, with error bars indicating the SEM. Expression of the indicated proteins was confirmed by western blotting

As the PACSIN2 SH3 domain is bound to proline‐rich motifs of both SH3BP1 and Cobll1, we compared the binding affinities of the three proteins using surface plasmon resonance (SPR) and nuclear magnetic resonance (NMR) studies. The SPR results indicated that the PACSIN2 SH3 domain bound to the N‐terminal region of Cobll1 (residues 1–410; Cobll1‐NT‐f) and the C‐terminal region of SH3BP1 containing all proline‐rich motifs (residues 541–701; SH3BP1‐CT) with dissociation constants (K_d_) of 25.8 nM and 2.2 μM, respectively. PACSIN2 SH3 domain exhibited an approximately 85‐fold higher binding affinity for the Cobll1‐NT‐f than for SH3BP1‐CT (Figure 4A,B). Through further NMR assessment, the ^1^H‐^15^N Heteronuclear single quantum coherence (HSQC) spectrum of the ^15^N‐labeled PACSIN2 SH3 domain in the presence of equimolar ratios of both Cobll1‐NT and SH3BP1‐CT (i.e., PACSIN2 SH3:Cobll1‐NT:SH3BP1‐CT = 1:1:1) was measured and compared to the spectra of PACSIN2 SH3 domain in complex with Cobll1‐NT or SH3BP1‐CT (Figure 4C). Because Cobll1 and SH3BP1 demonstrated similar binding patterns for the PACSIN2 SH3 domain (Figure S6), the majority of peaks for the PACSIN2 SH3 domain in the presence of Cobll1‐NT or SH3BP1‐CT overlapped. However, the chemical shifts of the nonoverlapping residues, such as G463, G467, L445, and E441 of the PACSIN2 SH3 domain, with both Cobll1‐NT and SH3BP1‐CT (green color) were much closer to those with Cobll1‐NT alone (red color) than to those with SH3BP1‐CT alone (blue color). These results suggest that the PACSIN2 SH3 domain is preferentially bound to Cobll1‐NT than to SH3BP1‐CT (Figure 4C).

The competitive binding of Cobll1 and SH3BP1 to PACSIN2 was further investigated by conducting a replacement titration (Figure S7A,B). The chemical shifts of the Y435, K455, Q462, and Q463 residues of the PACSIN2 SH3 domain bound to SH3BP1‐CT at the saturation point (blue color) were gradually shifted by the addition of increasing concentrations of Cobll1‐NT (Figure S7C, upper panel). By contrast, no (K455 and Q462) or a smaller (Y435 and G463) change in chemical shift was observed by the addition of SH3BP1‐CT to the Cobll1‐NT‐PACSIN2 complex (Figure S7C, lower panel). The replacement titration results were consistent with the PACSIN2 SH3 domain having a stronger binding affinity for Cobll1‐NT than SH3BP1‐CT. We further confirmed that the expression of the full‐length or the N‐terminus of Cobll1 inhibited the binding between PACSIN2 and SH3BP1 using immunoprecipitation assays (Figure 4D,E). Collectively, our data suggest that the PACSIN2 SH3 domain preferentially interacts with proline‐rich motifs of Cobll1, compared with SH3BP1.

### 3.6 PACSIN2 and SH3BP1 regulate myelopoiesis

**FIGURE 5:**
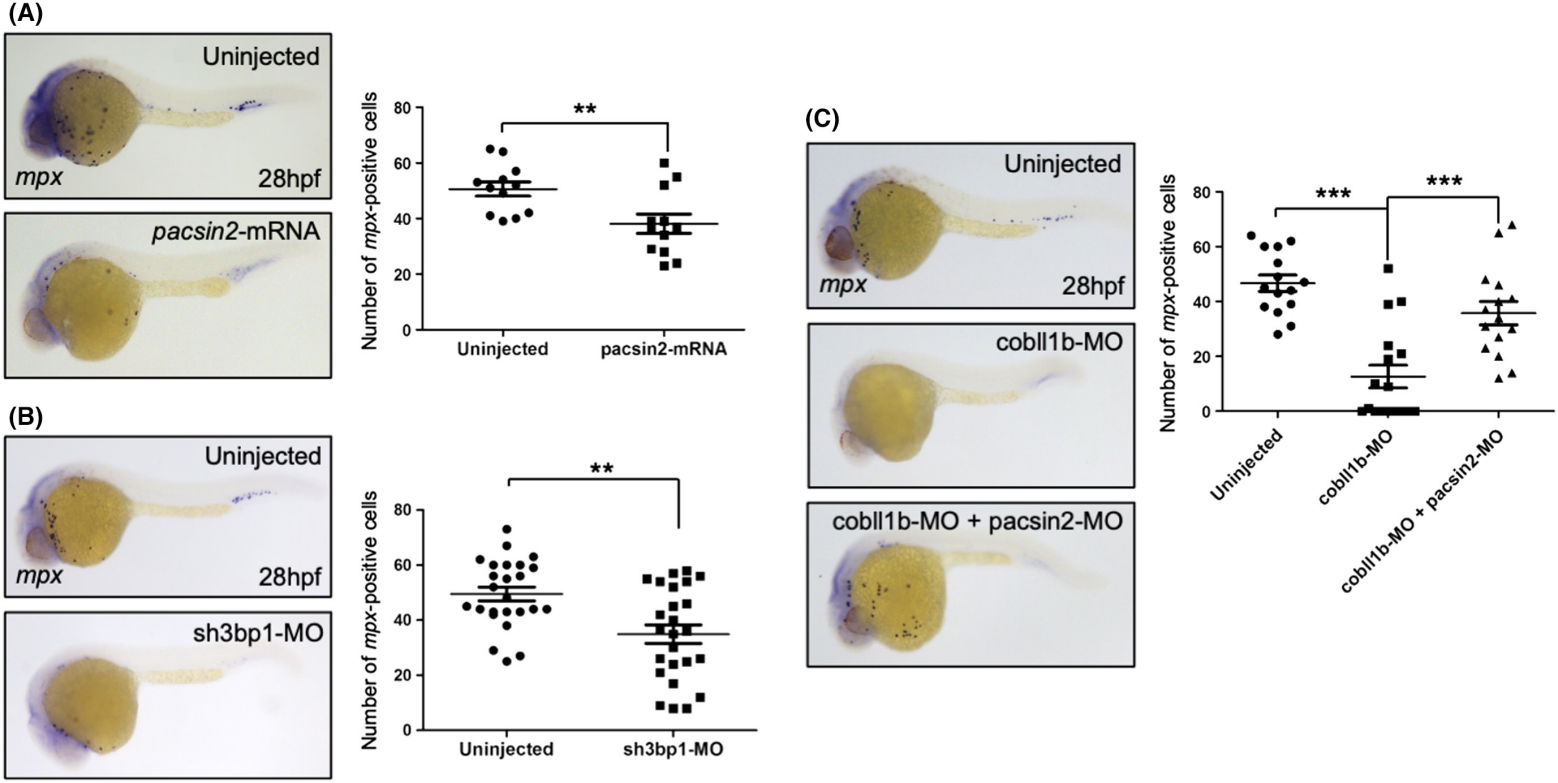
Pacsin2 and Sh3bp1 are required for normal hematopoiesis. (A and B) Images of *mpx* whole‐mount in situ hybridization (WISH) and the quantification of *mpx*‐positive cell population of embryos injected with *pacsin2* mRNA (28 hpf) (A: uninjected control; *n* = 12; pacsin2‐mRNA injected; n = 12) and sh3bp1‐MO (B: uninjected control, *n* = 25; sh3bp1‐MO, *n* = 25). (C) Images of *mpx* WISH of 28 hpf cobll1b‐MO and cobll1b‐MO and pacsin2‐MO co‐injected embryos and the quantification of *mpx*‐positive cells from cobll1b morphants and cobll1b + pacsin2 morphants compared with uninjected controls (uninjected control, *n* = 15; cobll1b‐MO, *n* = 15; cobll1b‐MO + pacsin2‐MO, *n* = 15). All error bars indicate the SEM. **P‐value <0.01, ***P‐value <0.001; significantly different from control, Student's *t*‐test

Due to the requirement of zebrafish *cobll1b* for normal myelopoiesis,^13^ we hypothesized that PACSIN2 and SH3BP1 are involved in hematopoiesis. To assess the function of PACSIN2 and SH3BP1 in myelopoiesis, we performed whole‐mount in situ hybridization using the myeloid marker *mpx* in zebrafish embryos at 28 hours postfertilization (hpf). Interestingly, the ectopic expression of *pacsin2* via mRNA injection diminished the number of *mpx*‐positive primitive myeloid cells. However, embryonic myelopoiesis was not altered in the *pacsin2* morphants, wherein *pacsin2* expression was inhibited by antisense morpholino (MO) oligonucleotides injection (Figure 5A and Figure S8A–C). Similarly, the morphants of both *sh3bp1* and *rac1a* had less myeloid cells than the controls (Figure 5B and Figure S8D). Collectively, these results suggest that the SH3BP1/Rac1a signaling cascade positively regulates myelopoiesis during zebrafish embryogenesis while PACSIN2 negatively regulates myeloid development.

Based on the binding studies showing that Cobll1 has a higher binding affinity to PACSIN2 than SH3BP1, we next hypothesized that endogenous Cobll1b constitutively binds to PACSIN2 to block its inhibition of the SH3BP1/Rac1a pathway during zebrafish myelopoiesis. To test this hypothesis, we co‐injected MOs against *cobll1b* and *pacsin2* to eliminate the effect of both *cobll1b* and *pacsin2*. As previously described, the cobll1b MO‐injected group showed a significant reduction in *mpx*‐positive cells at 28 hpf.^13^ By contrast, the simultaneous knockdown of *pacsin2* and *cobll1b* restored myeloid formation (Figure 5C), suggesting that the loss of *cobll1b* expression causes PACSIN2 to inhibit the SH3BP1/Rac1a pathway and leads to the perturbation of normal hematopoiesis. Taken together, these results suggested that a reciprocal regulatory network among Cobll1b, PACSIN2, and SH3BP1 controls embryonic hematopoiesis in vertebrates using the same mechanism that regulates nilotinib resistance in CML.

### 3.7 Clinical correlation of Cobll1/PACSIN2/SH3BP1 expression

**FIGURE 6:**
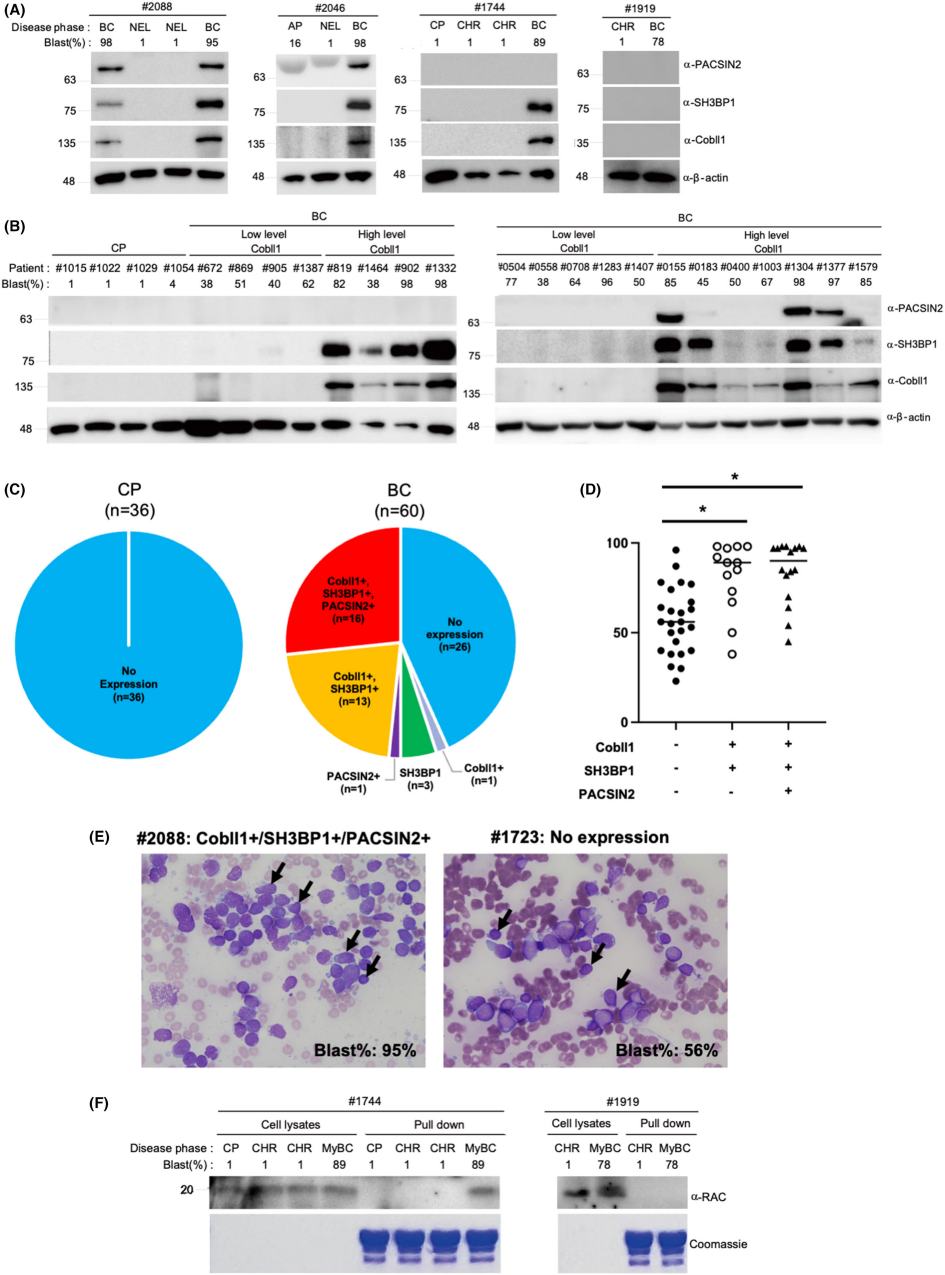
Clinical correlation of Cobll1/PACSIN2/SH3BP1 expression in patients with CML. (A and B) Images of western blot analyses examining the expression of Cobll1, PACSIN2, and SH3BP1 in the bone marrow mononuclear cells of paired serial samples (A) or unpaired samples (B) from patients with CML at CP and BC phases. (C) Pie charts illustrating the distribution of distinct expression patterns of Cobll1, PACSIN2, and SH3BP1 in patients with CML at the CP or BC phase, based on the western blot analyses. (D) Quantification of bone marrow blast percentage among Cobll1‐indepedent BC patients and BC patients with expression of Cobll1+/SH3BP1+ and Cobll1+/SH3BP1+/PACSIN2+. Cobll1−/SH3BP1−/PACSIN2− (*n* = 25); Cobll1+/SH3BP1+ (*n* = 13); Cobll1+/SH3BP1+/PACSIN2+ (*n* = 16). *P‐value <0.00005. (E) Representative images of May–Grunwald–Giemsa‐staining of bone marrow aspirate smears from BC patients with expression of Cobll1+/SH3BP1+/PACSIN2+ (#2088, blast%: 95%; left panel) and no expression (#1723, blast%: 56%; right panel). The blasts possess small‐ to medium‐sized nuclei with a coarse chromatin pattern and a scanty amount of cytoplasm. Original magnification, x400. Black arrows indicate the blasts. (F) GST pull‐down assays showed Rac1 activity in CP and BC samples from two patients (#1744 with expression of Cobll1 and SH3BP1, and #1919 with no expression of Cobll1, SH3BP1, or PACSIN2). Each sample number with the pound symbol (#) indicates the anonymous patient number from the Asia CML registry. AP, accelerated phase; BC: blast crisis; CHR, complete hematologic response; CP, chronic phase; MyBC, myeloid blast crisis; NEL, no evidence of leukemia

Previously, we showed that Cobll1 is highly upregulated in CML patients with BC progression, compared with those at the CP.^13^ Because Cobll1 activates the Rac1 pathway by regulating the PACSIN2–SH3BP1 interaction at the cellular and organismal level, we next investigated the correlated expression patterns among Cobll1, PACSIN2, and SH3BP1 in clinical samples. We first determined the expression levels of three proteins in the paired serial samples from 20 patients with CML. Interestingly, 13 patients at the BC phase showed positive expression with distinct combinatorial patterns of Cobll1/PACSIN2/SH3BP1 while no patients at the CP exhibited any expression of these three proteins (Figure 6A; Figure S9A). In addition, we further examined the expression of these proteins using the samples obtained from unpaired patient groups. Similar expression patterns of Cobll1/PACSIN2/SH3BP1 were observed in 18 patients among 31 BC patients, whereas no patients at the CP showed any expression of these proteins (Figure 6B; Figure S9B). Of 60 samples obtained from 51 BC patients, 36 showed the expression of Cobll1/PACSIN2/SH3BP1 in two major patterns, Cobll1+/SH3BP1+ (13/36), and Cobll1+/PACSIN2+/SH3BP1+ (16/36); however, the other 24 BC samples and all 36 CP samples showed no expression of any of these proteins (Figure 6C), suggesting that expression of both Cobll1 and SH3BP1 is linked to BC progression.

To further examine the correlation of Cobll1/PACSIN2/SH3BP1 with BC progression, we analyzed the clinical characteristics of 51 patients at the BC phase based on distinct combinatorial expression patterns of Cobll1/PACSIN2/SH3BP1 including bone marrow blast percentage. Interestingly, blast percentage was significantly higher in BC patients expressing Cobll1+/SH3BP1+ and Cobll1+/PACSIN2+/SH3BP1+ than in those with no expression (Cobll1−/PACSIN2−/SH3BP1−) (Table 1; Figure 6D). Representative images of Giemsa‐staining showed no morphological alteration in the blasts between the BC patients with either Cobll1+/PACSIN2+/SH3BP1+ or no expression, while the blast percentage is still higher in the patients expressing Cobll1+/PACSIN2+/SH3BP1+ (Figure 6E). The expression patterns of three proteins do not appear to be linked to the development of a specific type of BC‐CML between lymphoid BC and myeloid BC (Figure S9C). By contrast, the expression of BCR‐ABL1 showed no alteration between each patient group with different expression patterns (Table 1). In summary, these clinical data suggest that ectopic expression of Cobll1 and SH3BP1 can induce severe BC progression with higher blast percentage independent of BCR‐ABL.

**TABLE 1:** Clinical characteristics of CML patients at the BC phase with distinct expression patterns of Cobll1, PACSIN2, and SH3BP1

		G1	G2	G3	G4	G5	G6	
Parameters	All (*n* = 51)	Cobll1(+),PACSIN2(+),SH3BP1(+)(*n* = 13)	Cobll1(+),PACSIN2(−),SH3BP1(+)(*n* = 14)	Cobll1(+),PACSIN2(−),SH3BP1(−)(*n* = 1)	Cobll1(−),PACSIN2(+),SH3BP1(−)(*n* = 1)	Cobll1(−),PACSIN2(−),SH3BP1(+)(*n* = 2)	Cobll1(−),PACSIN2(−),SH3BP1(−)(*n* = 20)	*p* value
Age (years), median (range)	41 (9–77)	38 (9–61)	54 (16–77)	34	55	51.5 (50–53)	40 (13–68)	0.332
Sex, male, *n* (%)	34 (67)	10 (77)	8 (57)	0 (0)	1 (100)	0 (0)	15 (75)	0.140
Disease phase at diagnosis, *n* (%)								
CP/AP/BC	43 (84)/4 (8)/4 (8)	11 (84)/1 (8)/1 (8)	13 (93)/1 (7)/0 (0)	1 (100)/0 (0)/0 (0)	1 (100)/0 (0)/0 (0)	2 (100)/0 (0)/0 (0)	15 (75)/2 (10)/3 (15)	0.963
Transcript type, *n* (%)								
e13a2/e14a2/other(e14a2 + e1a2)	20 (39)/30 (59)/1 (2)	4 (30)/8 (62)/1 (8)	6 (43)/8 (57)/0 (0)	0 (0)/1 (100)/0 (0)	1 (100)/0 (0)/0 (0)	0 (0)/2 (100)/0 (0)	9 (45)/11 (55)/0 (0)	0.723
Type of BC								
LyBC/MyBC/unclassified BC	20 (39)/26 (51)/5 (10)	6 (46)/6 (46)/1 (8)	6 (43)/6 (43)/2 (14)	0 (0)/1 (100)/0 (0)	1 (100)/0 (0)/0 (0)	0 (0)/2 (100)/0 (0)	7 (35)/11 (55)/2 (10)	0.864
Blast (%), median (range)	78 (23–98)	97 (66–98)	87 (38–98)	70	95	40.5 (30–51)	60.5 (23–96)	0.000
BCR‐ABL1^IS^ transcript (%), median (range)	84.41308(11.32246–139.18450)	89.9368800(33.48214–95.26846)	86.7381050(55.31500–138.48806)	84.74897	104.13014	69.9931800(63.79953–76.18683)	76.095760(11.32246–139.18450)	0.516
BCR‐ABL1 mutation, *n* (%)								
Yes/no	32 (63)/19 (37)	5 (39)/8 (61)	10 (71)/4 (29)	1 (100)/0 (0)	1 (100)/0 (0)	2 (100)/0 (0)	13 (65)/7 (35)	0.292
SCT, *n* (%)								
Yes/no	17 (33)/34 (67)	6 (46)/7 (54)	4 (29)/10 (71)	1 (100)/0 (0)	0 (0)/1 (100)	0 (0)/2 (100)	6 (30)/14 (70)	0.453
Death, *n* (%)								
Yes/no	45 (88)/6 (12)	12 (92)/1 (8)	12 (86)/2 (14)	0 (0)/1 (100)	1 (100)/0 (0)	2 (100)/0 (0)	18 (90)/2 (10)	0.143

Abbreviations: BC, blast crisis; LyBC, lymphoid blast crisis; MyBC, myeloid blast crisis; IS, international scale; SCT, stem cell transplantation.

**FIGURE 7:**
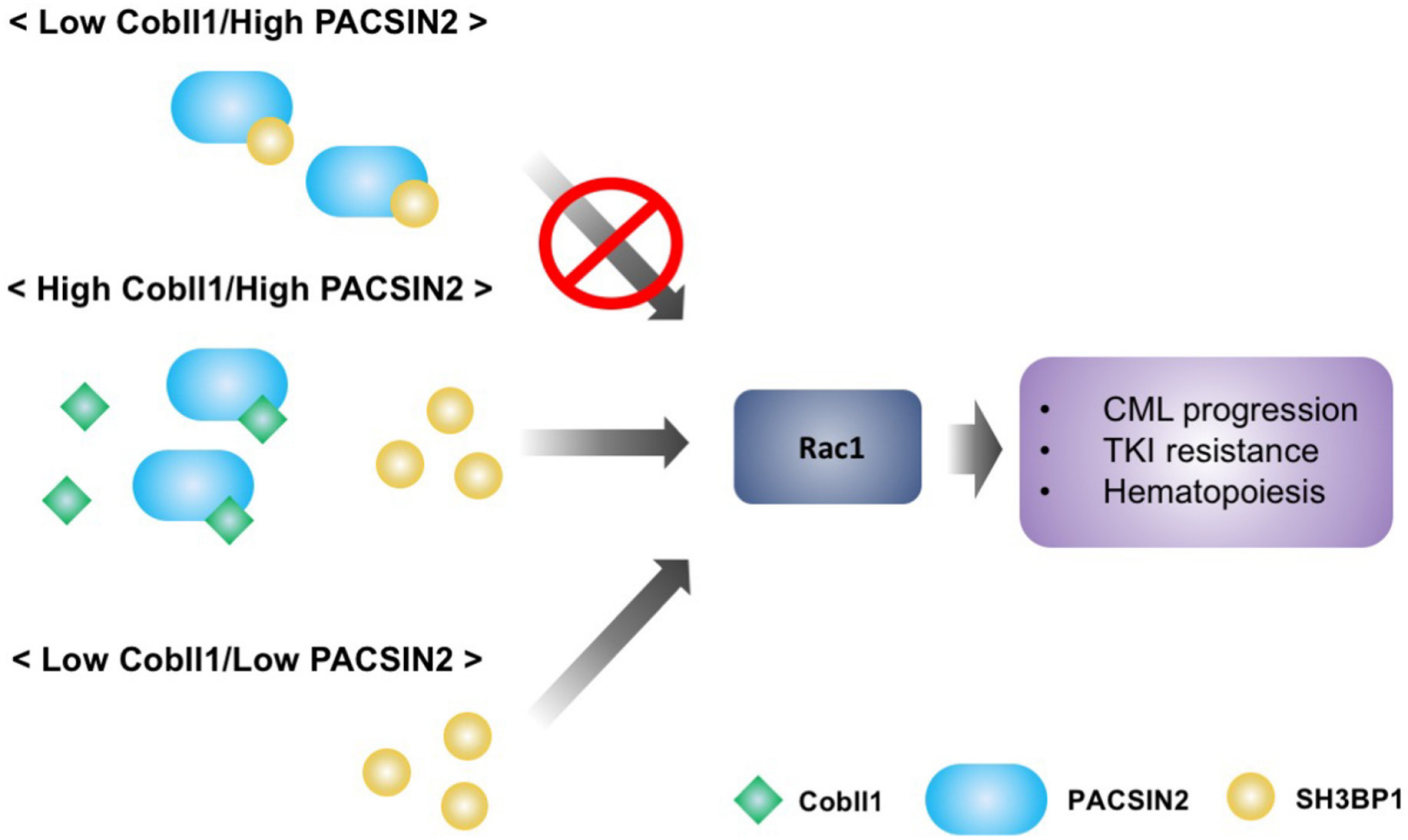
A cartoon model illustrating the reciprocal interactive mechanisms among Cobll1, PACSIN2, and SH3BP1 on hematopoiesis, drug resistance, and progression in CML

Finally, to confirm whether Rac1 is regulated by the Cobll1/PACSIN2/SH3BP1 pathway during CML progression, we examined the activity of Rac1 in samples from patients at the BC phase. We selected a patient sample (#1744) with Cobll1/SH3BP1 expression and a patient sample (#1919) without any Cobll1/PACSIN2/SH3BP1 expression in BC cells (Figure 6A). Consistent with primary cell and animal model studies, Rac1 activity was increased in BC cells of patient expressing Cobll1/SH3BP1 but not in patient without any Cobll1/PACSIN2/SH3BP1 expression, suggesting that the Rac1 pathway is only activated by an exclusive activation of both SH3BP1 and Cobll1 in patients with CML (Figure 6F). Taken together, our clinical analysis findings demonstrated that Cobll1/PACSIN2/SH3BP1 expression is correlated to BC progression in CML. In particular, Cobll1 and SH3BP1 activation leads to much poorer BC progression through the Rac1 pathway, independent of BCR/ABL1 (Figure 7).

## 4 DISCUSSION

CML development is generally caused by expression of BCR‐ABL1 fusion protein, which leads to genomic instability, and the malignant progression from the CP to the BC phase occurs in a BCR‐ABL1‐dependent or ‐independent manner.^35^, ^36^ Previously, our study revealed that the oncogenic protein Cobll1 is linked to TKI resistance and BC transformation in CML, independent of BCR‐ABL1 expression.^13^ In the present study, we found that the overexpression of PACSIN2, a novel functional binding partner of Cobll1, increased nilotinib sensitivity in K562 cells. The SH3 domain of PACSIN2 binds to the proline‐rich regions located at the N‐terminus of Cobll1, and both binding motifs are crucial for their antagonistic functions in TKI resistance. Likewise, the identical SH3 motif of PACSIN2 binds to the proline‐rich domains of SH3BP1, and SH3BP1 overexpression decreases the susceptibility of CML to nilotinib similar to the function of Cobll1. PACSIN2 interacts competitively with Cobll1 or SH3BP1 with higher affinity for Cobll1, and their reciprocal interaction eventually regulates TKI resistance, as well as normal hematopoiesis. More importantly, in the primary cells of 51 BC‐transformed patients with CML, the expression patterns of Cobll1, PACSIN2, and SH3BP1 were significantly associated with blast cell percentage. While none of the three proteins were expressed at the CP, more than half of BC patients showed expression of both Cobll1 and SH3BP1 with two representative patterns (Cobll1+/SH3BP1+ and Cobll1+/PACSIN2+/SH3BP1+) These data suggest that the interactions between Cobll1, PACSIN2, and SH3BP1 are closely linked to BC transformation, TKI resistance, and sustained hematopoiesis.

Although Cobll1 has been shown to be linked to diverse processes of cancer formation and progression including CML, CLL, and prostate cancer,^13^, ^14^, ^37^, ^38^ its molecular regulation has not been fully understood. Our study found that PACSIN2 plays a negative regulatory role against Cobll1 as a potential tumor suppressor in CML. Recent studies found that polymorphism of PACSIN2 is associated with drug‐induced hematotoxicity in patients with ALL undergoing therapy,^24^, ^39^ showing the potential effects of PACSIN2 on hematopoiesis suppression. Moreover, the activation of the Rac1 pathway leads to TKI resistance at the cellular level and promotes migration and self‐renewal of hematopoietic stem cells, participating in leukemia initiation and maintenance. SH3BP1 has been shown to activate Rac1 to regulate cancer cell behavior.^27^ Consistently, our data using K562 cell lines showed that SH3BP1 activated the Rac1 signaling pathway, whereas PACSIN2 inhibited Rac1 activity through its interaction with SH3BP1. The overexpression of Cobll1, which preferentially binds PACSIN2, forces SH3BP1 to be released from its complex with PACSIN2, activating the Rac1 signaling pathway to induce TKI resistance. In patients with CML, Rac1 activity was robustly higher in the BC sample (#1744) expressing Cobll1 and SH3BP1 than in the BC sample (#1919) without the expression of either Cobll1 or SH3BP. In summary, the Rac1 activation by the Cobll1/PACSIN2/SH3BP1 interactive cascade is closely linked to drug resistance and BC progression in CML.

In the clinical samples, three expression patterns of Cobll1, PACSIN2, and SH3BP1 were primarily observed: Cobll1+/SH3BP1+, Cobll1+/PACSIN2+/SH3BP1+, and none. By contrast, no CP patients showed any expression of the three proteins, suggesting that these proteins are related to BC progression. In BC patients, the expression of Cobll1 alone did not induce BC progression, but the co‐expression of SH3BP1, which activates the Rac1 pathway, appears to activate more aggressive BC progression. In addition, PACSIN2‐expressing BC samples without Cobll1 were rarely observed, possibly due to the negative effects of PACSIN2 on SH3BP1. Furthermore, BC patients with Cobll1+/SH3BP1+ or Cobll1+/PACSIN2+/SH3BP1+ showed a higher blast percentage than those without any expression of the three proteins, indicating that Cobll1 and SH3BP1 can aggravate disease progression in CML. Although the expression of both Cobll1 and SH3BP1 is highly correlated to the severity of BC progression with higher blast percentage, the transcript level of BCR‐ABL1 was not significantly correlated with the expression of both proteins. These results demonstrate that Cobll1/SH3BP1‐dependent CML progression is independent of BCR‐ABL1 and that the Cobll1/PACSIN2/SH3BP1 regulatory cascade can be a novel target for drug resistance and disease progression in CML in a BCR‐ABL1‐independent manner. Taken together, our cellular and organismal studies of Cobll1/PACSIN2/SH3BP1 and clinical analysis of patient samples showed that the Cobll1/SH3BP1/PACSIN2 axis that modulates Rac1 activity regulates drug resistance and disease progression in CML.

## CONFLICT OF INTEREST

The authors declare that they have no competing interest.

## AUTHOR CONTRIBUTIONS

M.D.S., Y.L., S.H.H., H.K., and D.W.K. designed the experiments; K.P., H.Y., C.O., J.R.L., H.J.C., H.K., and S.K. performed the experiments; M.D.S., Y.L., S.H.H., H.K., and D.W.K. analyzed the data and wrote the manuscript.

## ETHICS APPROVAL AND CONSENT TO PARTICIPATE

All human samples were obtained from the Korea Leukemia Bank and the protocol was approved by the Institutional Review Board (IRB) of the Songeui Campus, Catholic University of Korea (KIRB‐0E481‐001). Patient consent was obtained in accordance with the Declaration of Helsinki.

## Supporting information


Data S1

## Data Availability

All data generated or analyzed during this study are included in this published article and its supplementary information files.
