# Supplementary material for: Reciprocal interactions among Cobll1, PACSIN2, and SH3BP1 regulate drug resistance in chronic myeloid leukemia
Source: Cancer Med. 2022 Mar 30;11(21):4005–20. doi: 10.1002/cam4.4727 (PMC9636508; doi:10.1002/cam4.4727)
Supplement: Supplementary file 1 — Data S1 [file CAM4-11-4005-s001.docx]

**Supplementary materials and methods**

**Mass spec analysis**

For LC-MS/MS analyses, the coommassie stained gel was de-stained and bands cut and processed as follows. Briefly, isolated proteins bands were divided into 10 mm sections and subjected to in-gel digestion with trypsin. The tryptic digests were separated by online reversed-phase chromatography using a Thermo Scientific Eazy nano LC 1200 UHPLC equipped with an autosampler using a reversed-phase peptide trap Acclaim PepMapTM 100 (75 μm inner diameter, 2 cm length) and a reversed-phase analytical column PepMapTM RSLC C18 (75 μm inner diameter, 15 cm length, 3 μm particle size), both from Thermo Scientific, followed by electrospray ionization at a flow rate of 300 nl min−1. Samples were eluted using a split gradient of 3-50% solution B (80% ACN with 0.1% FA) in 60 min and 50-80% solution B in 10 min followed column wash at 100% solution B for 10 min. The chromatography system was coupled in line with an Orbitrap Fusion Lumos mass spectrometer. The mass spectrometer was operated in a data-dependent mode with the 120,000 resolution MS1 scan (375-1500 m/z), AGC target of 5e5 and max injection time of 50ms. Peptides above threshold 5e3 and charges 2-7 were selected for fragmentation with dynamic exclusion after 1 time for 15 s and 10 ppm tolerance. Spectra were searched against the uniprot-human DB using the Proteome Dsciverer Sorcerer 2.1 with SEQUEST-based search algorithm, and comparative analysis of proteins identified in this study was performed using Scaffold 4 Q+S.

**siRNAs and transfection.**

All siRNA duplexes of Cobll1 and PACSIN2 were purchased from IDT (Integrated DNA Technologies Inc., Coralville, IA, USA). The sequences are as follows:

Cobll1 siRNA1 sense: 5′-CUAUGAUGGACUUGUUGAUUU-3′

Cobll1 siRNA1 antisense: 5′-AUCAACAAGUCCAUCAUAGUU-3′

Cobll1 siRNA2 sense: 5′-GACUCAUUCUGUAAAUAAAUU-3′

Cobll1 siRNA2 antisense: 5′-UUUAUUUACAGAAUGAGUCUU-3′

PACSIN2 siRNA1 sense: 5′-CAAAUUAUGUGGAGGCGAUUU-3′

PACSIN2 siRNA1 antisense: 5′-AUCGCCUCCACAUAAUUUGUU-3′

PACSIN2 siRNA2 sense: 5′-GGAGAAGCUGGCUAUCUCACGAGAAUU-3′

PACSIN2 siRNA2 antisense: 5′-UUCUCGUGAGAUAGCCAGCUUCUCCUU-3′

SH3BP1 siRNA1 sense: 5′-UCAUGAUGCUGCUUUCUGAUU-3′

SH3BP1 siRNA1 antisense: 5′-UCAGAAAGCAGCAUCAUGAUU-3′

SH3BP1 siRNA2 sense: 5′-GACAUCAACUUCAACGUGUUU-3′

SH3BP1 siRNA2 antisense: 5′-ACACGUUGAAGUUGAUGUCUU-3′

All siRNAs and plasmids were transiently transfected into cells using electroporation. Specifically, K562 cells (6 × 10^6^ or 2 × 10^6^ cells) were electroporated using the Neon Transfection System (MPK10096, Invitrogen, Carlsbad, CA, USA). A total of 20 μg plasmid DNA or 200 nM siRNA was added to the cells and electroporation was performed according to the manufacturer’s protocol (1450 V for 15 ms in 2 pulses), and the transfected cells were incubated in prewarmed RPMI medium supplemented with 10% FBS for 48 h.

**GST pull-down.**

The GST fusion protein or GST was expressed in *Escherichia coli* and immobilized on glutathione-Sepharose 4B beads. Cell lysates of HEK293T cells transfected with plasmids encoding the indicated proteins were incubated with the GST fusion protein or GST for one hour at 4°C. After washing with NETN buffer, the samples were subjected to SDS-PAGE. Western blotting was performed using the antibodies indicated in the ﬁgure legends.

**NMR spectroscopy**

For Cobll1-NT (residues 175–370) and SH3PB1-CT replacement titrations, unlabeled Cobll1-NT and SH3BP1-CT proteins were purified and prepared in the same buffer (50 mM sodium phosphate, 100 mM NaCl, 1 mM TCEP, pH 6.3) at concentrations from 1 mM to 2.2 mM. The proteins were titrated to 50 μM of ^15^N-labeled PACSIN2 SH3 domain at various molar ratios (PACSIN2 SH3 domain:Cobll1-NT or SH3BP1-CT = 1:0, 1:0.25, 1:0.5, 1:1, 1:2). In the replacement titration, unlabeled Cobll1-NT proteins were added to the ^15^N-labeled PACSIN2 SH3 in the presence of SH3BP1 at various molar ratios (PACSIN2 SH3 domain:SH3BP1-CT:Cobll1-NT = 1:1:0.1, 1:1:0.25, 1:1:0.5, 1:1:0.75, 1:1:1, 1:1:2), and vice versa. The CSPs were calculated using the following equation: CSP (∆δ)= $\sqrt{\left( \Delta\delta_{H} \right)^{2}+1/6\left( \Delta\delta_{N} \right)^{2}}$, where Δδ_H_ and Δδ_N_ are the amide proton and nitrogen chemical shift differences, respectively (Williamson, 2013). All spectra were processed using NMRpipe and visualized using NMRViewJ software.

**Whole-mount in situ hybridization (WISH)**

Embryos were fixed at 28 hpf with 4% paraformaldehyde in PBS overnight at 4°C, and then dehydrated with methanol at −20°C overnight. The dehydrated embryos were rehydrated with PBS-0.1% Tween-20 (PBT) solution and permeabilized using acetone at −20°C. The permeabilized embryos were prehybridized with hybridization buffer (50% formamide, 5× SSC, 500 μg/ml torula yeast tRNA, 50 μg/ml heparin, 0.1% Tween-20, and 9 mM citric acid (pH 6.5)) for one hour at 68°C, and then were hybridized with a digoxigenin (DIG)-labeled antisense RNA probe for *mpx* in hybridization buffer for 3 days at 68°C. After washing the samples with hybridization buffer, 2× SSC, 0.2× SSC, and PBT solution, they were incubated with alkaline phosphatase-conjugated anti-DIG antibodies (1:4000) (Roche, Mannheim, Germany) overnight at 4°C. The samples were then washed six times with PBT solution and incubated in AP buffer (100 mM Tris, pH 9.5, 50 mM MgCl_2_, 100 mM NaCl, and 0.1% Tween-20) with NBT/BCIP substrate (Promega, Madison, USA) to visualize the WISH signals.

**Supplementary Figures and Figure Legends**


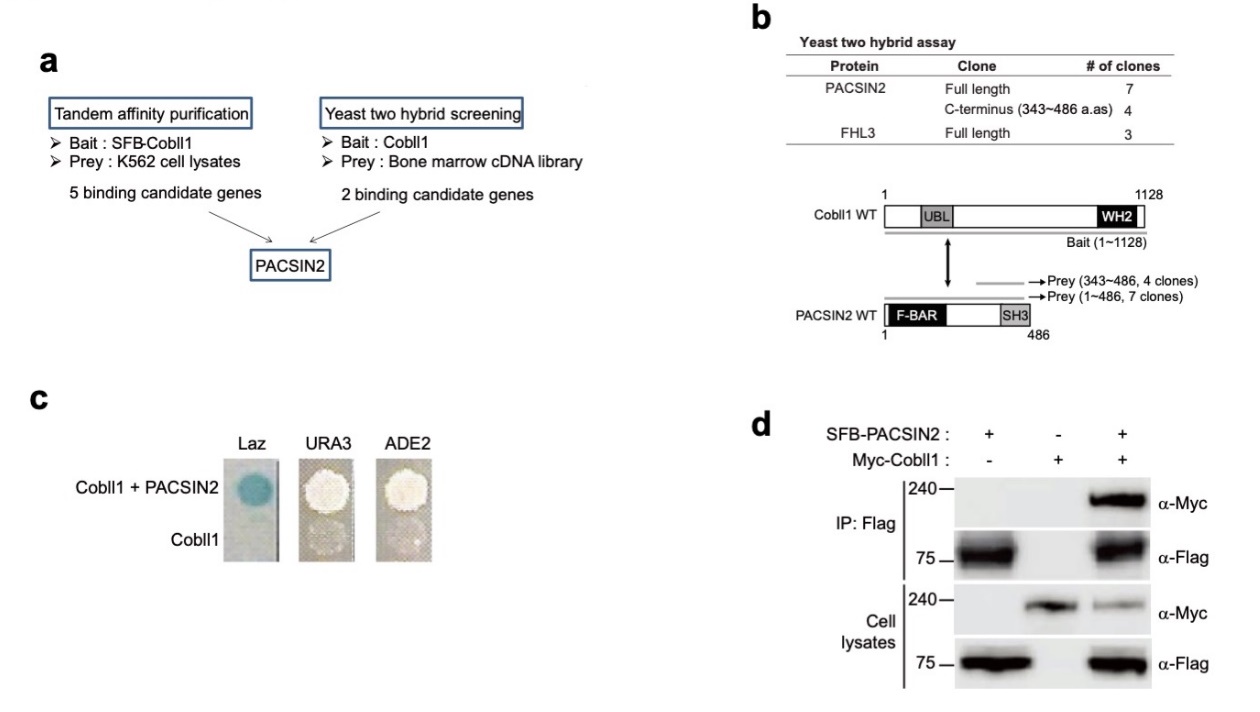


**Figure S1.** Finding binding proteins of Cobll1. (**A**) Schematic diagram of mass spectrometry and yeast two-hybrid assay for purification. (**B**) Proteins identified by yeast two-hybrid analyses of the Cobll1 protein (upper) and schematic structure of Cobll1 and PACSIN2 (lower). The gray line below Cobll1 indicates the bait, and the two gray lines above PACSIN2 highlight the prey clones identified in the yeast two-hybrid screening. (**C**) Indicated protein expression vectors were co-transformed into yeast AH109 cells and growing colonies were assessed on high stringent media. Blue colonies on the selective media indicate a positive interaction. (**D**) Interaction between overexpressed Cobll1 and PACSIN2 in K562 cells.

**
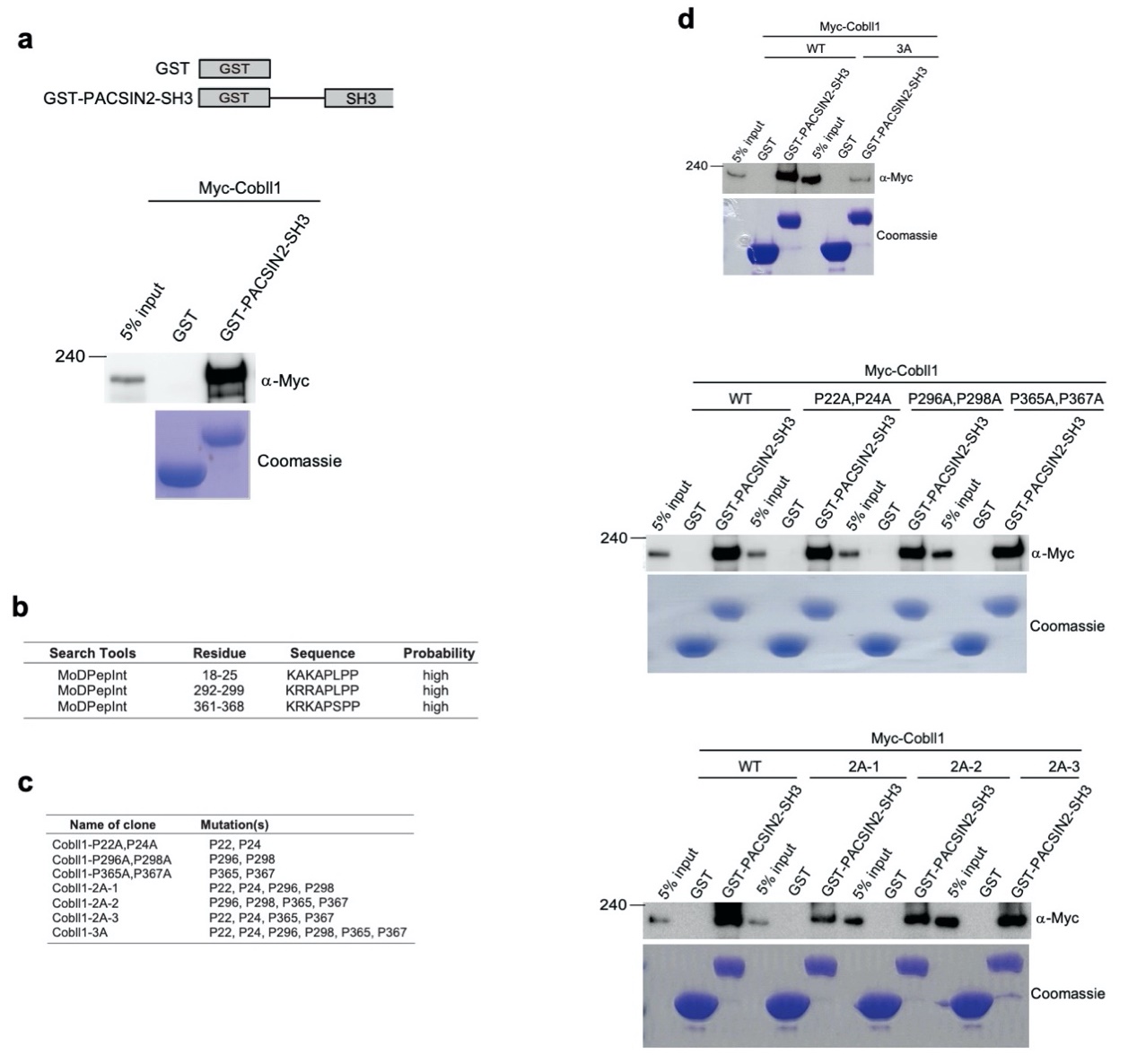
**

**Figure S2.** SH3 domain of PACSIN2 binds proline-rich regions of Cobll1 protein. (**A**) Diagram of GST-PACSIN2 SH3 and GST-pull down assay showing the interaction between GST-PACSIN2 SH3 and overexpressed Cobll1. (**B**) Potential proline-rich regions in the Cobll1 protein predicted by MoDPepint server. (**C**) Summary of mutants of proline-rich regions in Cobll1. Key proline residues have been mutated to alanine. Cobll1-2A and Cobll1-3A indicate point mutants where two and three proline-rich motifs of Cobll1 have been mutated, respectively. (**D**) Interactions between GST-PACSIN2 SH3 and overexpressed mutants of proline-rich regions in Cobll1.


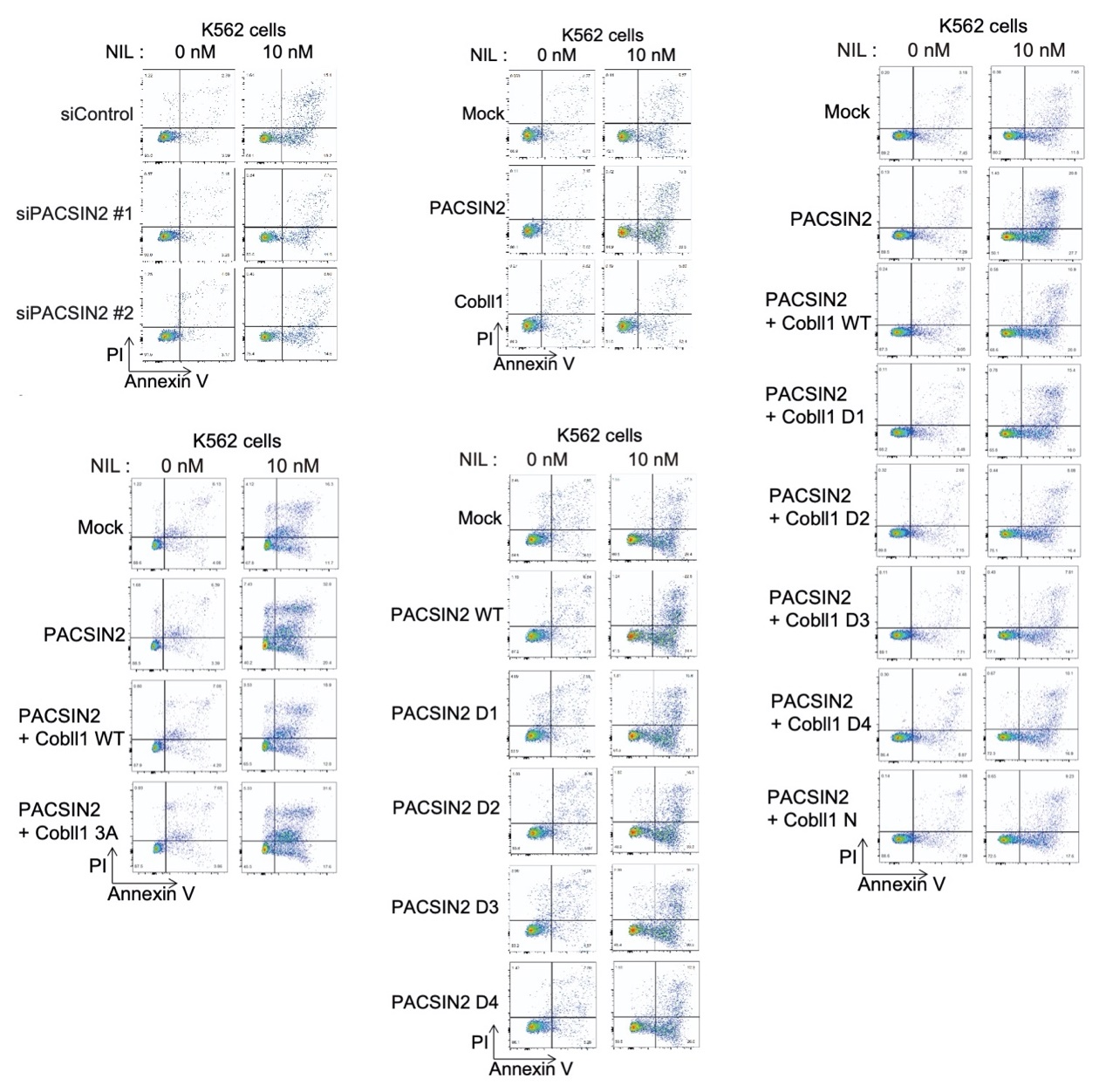
 **Figure S3.** Nilotinib-induced apoptosis assays using FACS analysis. FACS analysis was used for the validation of apoptotic events in K562 cells with siRNAs against PACSIN2 and the overexpression of the indicated genes.

**
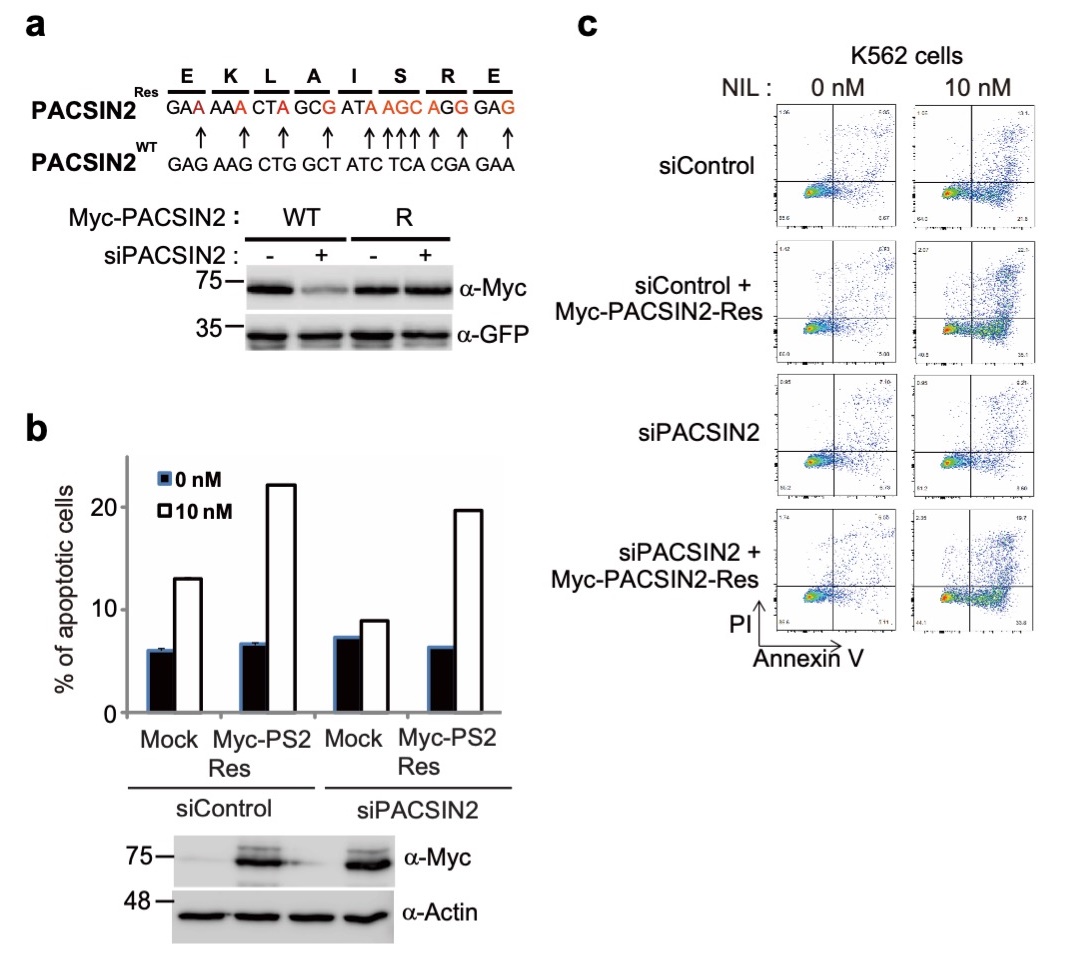
 Figure S4.** PACSIN2 siRNA is specific to target PACSIN2 mRNA. (**A**) Diagram of siRNA-resistant Myc-PACSIN2 plasmid and a western blot image for wild-type Myc-PACSIN2 and siRNA-resistant Myc-PACSIN2 with PACSIN2 siRNA treatment. (**B**) Bar graph illustrating the percentage of apoptotic cells. Expression of siRNA-resistant Myc-PACSIN2 in K562 cells rescues nilotinib-induced apoptosis. (**C**) FACS analysis for validation of apoptotic events in K562 cells using siRNA against PACSIN2 with the expression of siRNA-resistant Myc-PACSIN2.

**
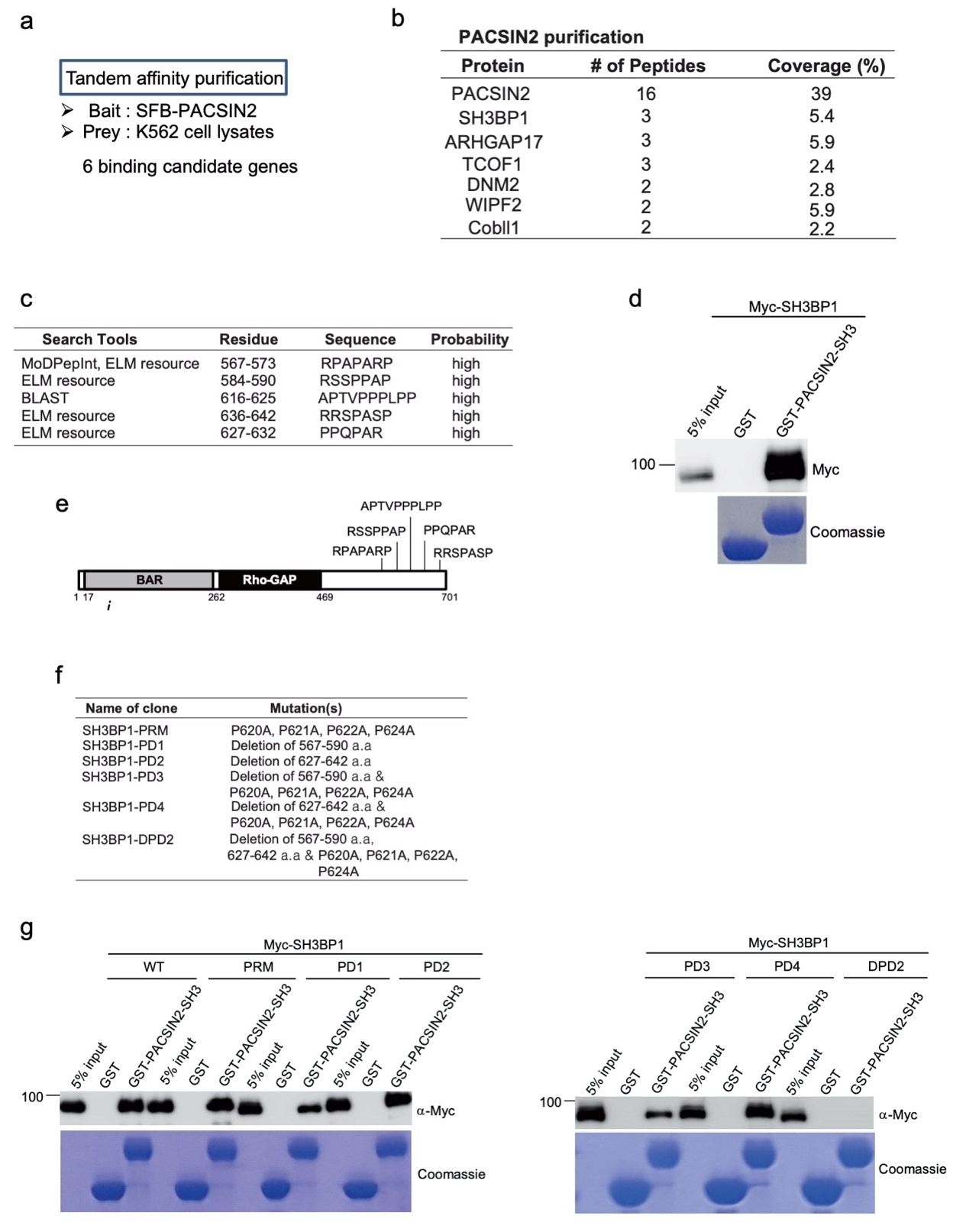
**

**Figure S5.** SH3BP1 binds to the SH3 domain of PACSIN2. (**A**) Purification scheme for mass spectrometry to identify PACSIN2 binding proteins. (**B**) Proteins identified by mass spectrometric analyses. (**C**) Predicted proline-rich regions of SH3BP1 using the MoDPepInt server, the Eukaryotic Linear Motif resource, and BLAST. (**D**) GST-pulldown assay showing the interaction between GST-PACSIN2 SH3 and overexpressed SH3BP1. (**E**) Diagram of predicted proline-rich regions within SH3BP1. (**F**) Summary of mutants of proline-rich regions in SH3BP1. Key proline residues have been mutated to alanine (PRM: proline-rich motif) or selected regions have been deleted (PD: PRM deletion). SH3BP1-DPD2 indicates a deletion mutant wherein all five proline-rich motifs of SH3BP1 have been deleted. (**G**) Interactions between GST-PACSIN2 SH3 and the overexpressed mutants of proline-rich regions in SH3BP1.


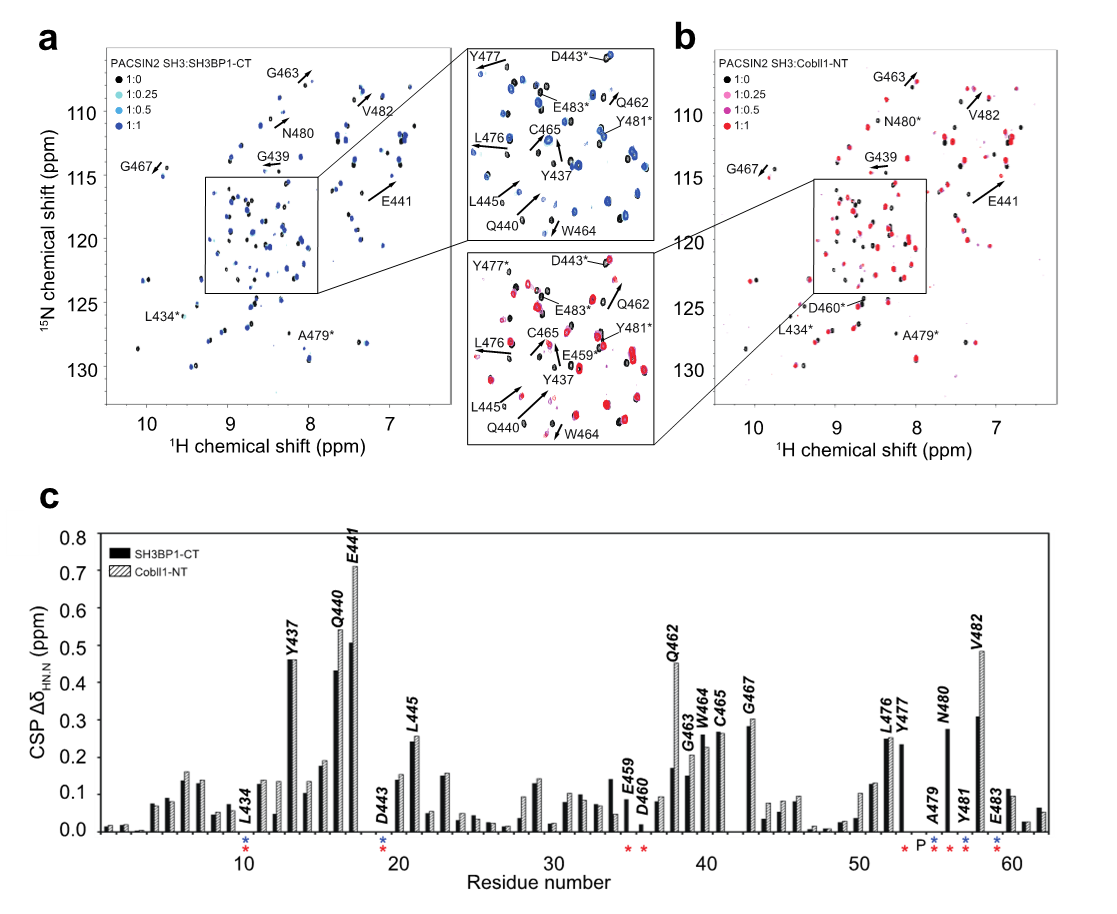


**Figure S6**. Heteronuclear single quantum coherence (HSQC) spectroscopy spectra of the PACSIN2 SH3 domain in the presence of SH3BP1-CT (**A**) and Cobll1-NT (**B**). Crowded regions have been enlarged, highly affected residues have been marked, and residues with broadened signals have been indicated with asterisks. (**C**) Chemical shift perturbations (CSP) values of PACSIN2 SH3 domain upon binding with SH3BP1-CT and Cobll1-NT. Residues that disappeared in the presence of SH3BP1-CT and Cobll1-NT are indicated by blue and red asterisks, respectively.

**
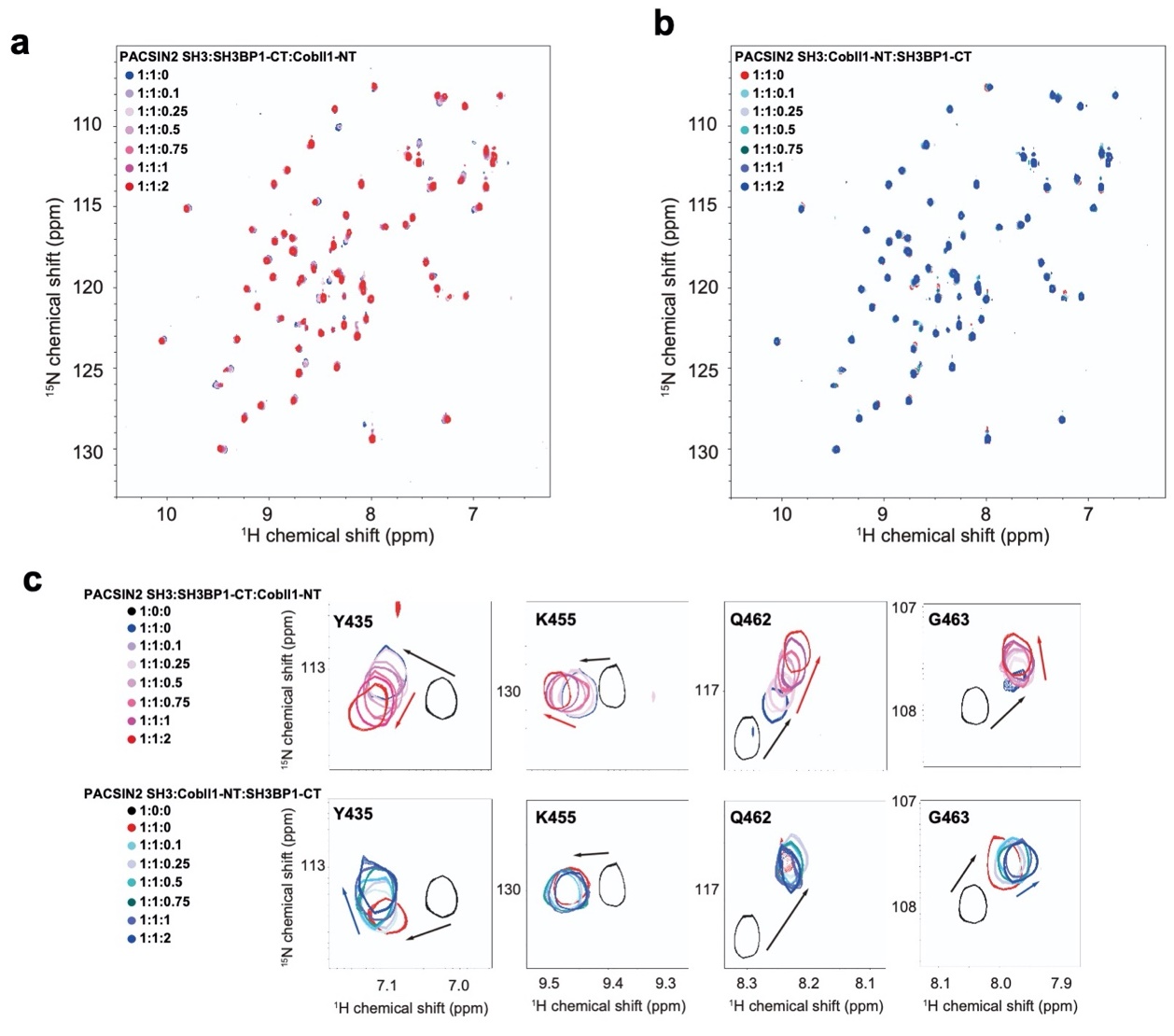
**

**Figure S7**. PACSIN2 competes for binding with SH3BP1 and Cobll1 through the SH3 domain. (**A** and **B**) Varied concentrations of Cobll1-NT titrated to the ^15^N-labeled PACSIN2 SH3 domain in a complex with SH3BP1-CT (**C**) and vice versa (**D**). In this experiment, SH3BP1-CT was added to the ^15^N-labeled PACSIN2 SH3 domain (PACSIN2 SH3:SH3BP1-CT, 1:1), followed by a titration of Cobll1-NT at various molar ratios and vice versa. (**C**) Varied concentrations of Cobll1-NT titrated to the ^15^N-labeled PACSIN2 SH3 domain in a complex with SH3BP1-CT (PACSIN2 SH3:SH3BP1-CT, 1:1) (upper) and vice versa (lower). The selected four residues (Y435, K455, Q462, and G463) are highlighted. Black arrows represent the chemical shift changes in the presence of one equivalent of SH3BP1-CT (upper) or Cobll1-NT (lower). Continuous chemical shift changes by adding the increasing concentrations of Cobll1-NT and SH3BP1-CT are denoted by red and blue arrows, respectively.


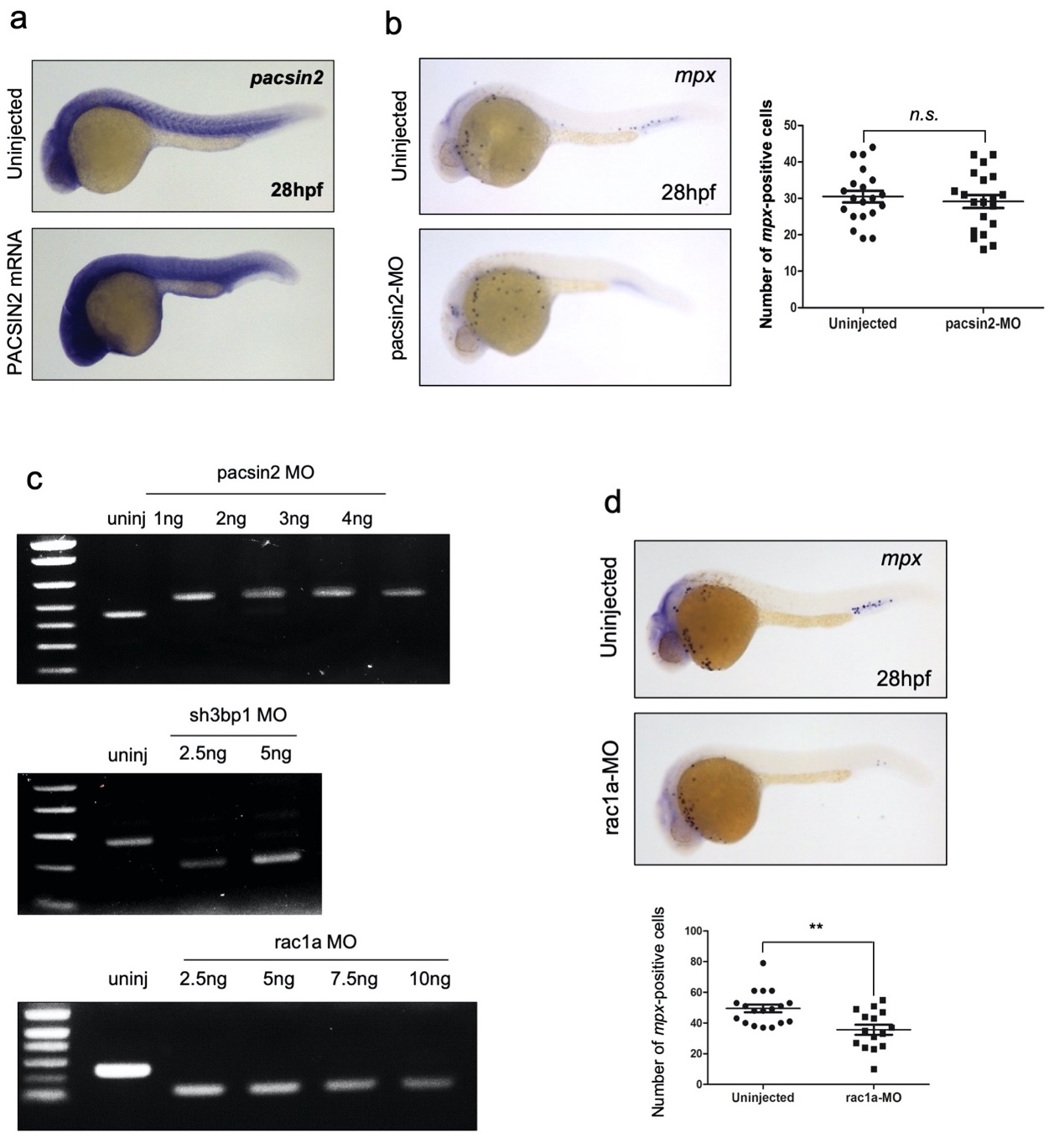


**Figure S8.** Pacsin2 and Sh3bp1 regulates myelopoiesis. (**A**) Images of *pacsin2* WISH in embryos injected with *pacsin2* mRNA at 28 hpf and uninjected controls. (**B**) Images of *mpx* WISH in embryos injected with pacsin2-MO at 28 hpf and controls, and the quantification of *mpx*-positive cell population in pacsin2 morphants (uninjected control, n = 20; pacsin2-MO, n = 20). (**C**) RT-PCR analysis using the cDNA from 28-hpf zebrafish embryos of uninjected controls (uninj) and embryos injected with each designated splice-blocking MO with varied doses. The targeted intron was included by pacsin2-MO injection (wild-type *pacsin2* amplicon, 450 bp; morphant amplicon, 565 bp) while targeted exons were successfully skipped in both *sh3bp1* morphants (wild-type *sh3bp1* amplicon, 275 bp; morphant amplicon, 196 bp) and *rac1a* morphants (wild-type *rac1a*, 255 bp; morphant amplicon, 137 bp), resulting in a frame-shift mutation causing a premature stop codon in each transcript. (**D**) Images of *mpx* WISH and the quantification of *mpx*-positive cell population of 28-hpf embryos injected with rac1a-MO (uninjected control, n = 18; rac1a-MO, n = 15). n.s., P-value > 0.05, not significantly different from control; *** P-value < 0.001, significantly different from control, Student’s t-test.

**
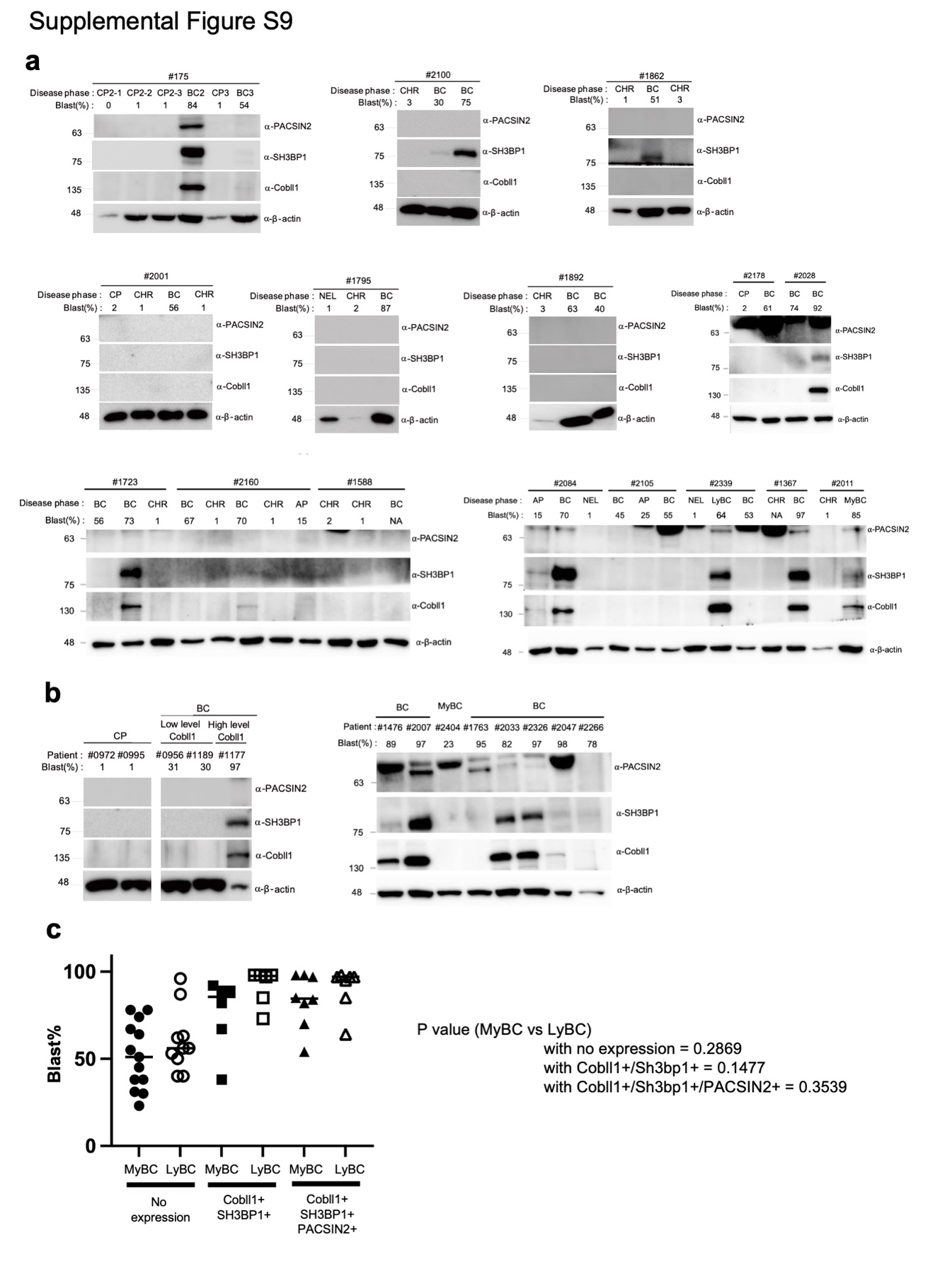
**

**Figure S9.** Clinical correlation of Cobll1/PACSIN2/SH3BP1 expression in the patients with CML. (**A** and **B**) Additional western blot analyses illustrating the expression pattern of Cobll1, PACSIN2, and SH3BP1 in the bone marrow mononuclear cells of paired serial samples (**A**) and unpaired samples (**B**) from patients with CML at CP and BC phases. Each sample number with the pound symbol (#) indicates the anonymous patient number from the Asia CML registry. AP, accelerated phase; CHR, complete hematologic response; MyBC, myeloid blast crisis; NEL, no evidence of leukemia. (**C**) Quantification of blast percentage of the bone marrow samples from myeloid BC (MyBC) and lymphoid BC (LyBC) patients with the expression of Cobll1+/SH3BP1+, Cobll1+/SH3BP1+/PACSIN2+, and no expression. All P-values between MyBC and LyBC were higher than 0.14, indicating no significant differences between MyBC and LyBC.

**Supplementary Reference**

Williamson MP (2013) Using chemical shift perturbation to characterise ligand binding. *Progress in nuclear magnetic resonance spectroscopy* 73: 1-16
